# The effect of randomised exposure to different types of natural outdoor environments compared to exposure to an urban environment on people with indications of psychological distress in Catalonia

**DOI:** 10.1371/journal.pone.0172200

**Published:** 2017-03-01

**Authors:** Margarita Triguero-Mas, Christopher J. Gidlow, David Martínez, Jeroen de Bont, Glòria Carrasco-Turigas, Tania Martínez-Íñiguez, Gemma Hurst, Daniel Masterson, David Donaire-Gonzalez, Edmund Seto, Marc V. Jones, Mark J. Nieuwenhuijsen

**Affiliations:** 1 ISGlobal, Centre for Research in Environmental Epidemiology (CREAL), Barcelona, Spain; 2 Universitat Pompeu Fabra (UPF), Barcelona, Spain; 3 CIBER Epidemiología y Salud Pública (CIBERESP), Barcelona, Spain; 4 Centre for Sport Health and Exercise Research, Staffordshire University, Stoke-on-Trent, United Kingdom; 5 Physical Activity and Sports Sciences Department, Fundació Blanquerna, Ramon Llull University, Barcelona, Catalonia, Spain; 6 Department of Environmental and Occupational Health Sciences, School of Public Health, University of Washington, Seattle, United States of America; Public Library of Science, FRANCE

## Abstract

**Introduction:**

Experimental studies have reported associations between short-term exposure to natural outdoor environments (NOE) and health benefits. However, they lack insight into mechanisms, often have low external and ecological validity, and have rarely focused on people with some psycho-physiological affection. The aim of this study was to use a randomized, case-crossover design to investigate: (i) the effects of unconstrained exposure to real natural and urban environments on psycho-physiological indicators of people with indications of psychological distress, (ii) the possible differential effects of 30 and 30+180 minutes exposures, and (iii) the possible mechanisms explaining these effects.

**Material and methods:**

People (n = 26) with indications of psychological distress were exposed to green (Collserola Natural Park), blue (Castelldefels beach) and urban (Eixample neighbourhood) environments in Catalonia. They were exposed to all environments in groups for a period of 30+180 minutes between October 2013 and January 2014. During the exposure period, participants were instructed to do what they would usually do in that environment. Before, during (at 30 and 30+180 minutes) and after each exposure, several psycho-physiological measures were taken: mood (measured as Total Mood Disturbance, TMD), attention capacity (measured as backwards digit-span task), stress levels (measures as salivary cortisol), systolic and diastolic blood pressure, heart rate, autonomous nervous system (assessed as heart rate variability and the indicators: low frequency power (LF), high frequency power (HF), ratio between LF and HF (LF:HF), and coefficients of component variance of LF, HF, and LF:HF). We also measured several potential mediators: air pollution, noise, physical activity, social interactions, and self-perceived restoration experience.

**Results:**

When compared with responses to urban environment, we found statistically significantly lower TMD [-4.78 (-7.77, -1.79) points difference], and salivary cortisol [-0.21 (-0.34, -0.08) log nmol/L] in the green exposure environment, and statistically significantly lower TMD [-4.53 (-7.57, -1.49) points difference], and statistically significant favourable changes in heart rate variability indicators (specifically LF:HF and CCV-LF:HF with around -0.20 points of difference of the indicators) in the blue exposure environment. Physical activity and self-perceived restoration experience partially mediated the associations between NOE and TMD. Physical activity and air pollution partially mediated the associations between NOE and heart rate variability.

**Discussion and conclusions:**

This study extends the existing evidence on the benefits of NOE for people's health. It also suggests NOE potential as a preventive medicine, specifically focusing on people with indications of psychological distress.

**Trial registration:**

Clinicaltrials.gov NCT02624921

## Introduction

In experimental studies, short-term exposure to NOE has been associated with well-being and health benefits, reporting psycho-physiological benefits that include: positive mood[1–5], improved attention[4,6], increased self-esteem[1,2,6], greater heart rate variability[2,3,5,7,8], reduced heart rate[2,4,5,7], and reduced blood pressure[2–7] (these last two indicators considered benefits when their reduction implies a reversion of elevated rates to healthy levels).

Various mechanisms have been suggested to explain the association between NOE and health and well-being. These include: increased opportunities for physical activity and social interaction, stress reduction and restorative effects, and reduced exposure to environmental pollutants, such as noise and air pollution[9,10]. However, there is not yet a clear understanding of these mechanisms, which have rarely been studied together or in experimental studies[3,8,10–15].

Apart from the lack of insight into mechanisms, there are other limitations in existing experimental studies. A number of studies have used students[3,5,6] or used opportunistic sampling methods[1,3,5–8,15,16], and many have only included participants of one sex[3,5,8], which limits their external validity. Also, the existing studies are frequently highly controlled experiments[2–7,15,16], focusing on the associations after induced stress[2,8], and using laboratory settings[2,7,8]. Therefore, ecological validity of existing experimental evidence is likely to be low.

Other limitations in the existing literature include the fact that the effects of NOE in populations with some psycho-physiological affection. have rarely been studied, with the exception of Barton et al. (2012) who studied the effects of exposure to NOE on people with mental health problems (i.e. substance-related, psychotic, mood, or anxiety disorders) and Sonntag-Öström et al. (2014) who studied exposure to NOE on people with exhaustion disorders. Also, few studies have investigated the effects of long exposures[17–20]. Finally, few have studied the effects of blue environments (those that include water bodies), with the exception of a study that included a swimming pool[17], one that included a waterside[1], one that included a forest with a path leading to a stream[8], one that included a forest next to a lake[4], one that included a vegetated footpath besides a canal[16] and one that included seacoasts and estuaries[21]. However, these investigating blue environments have indicated towards a psycho-physiological benefit of them.

We hypothesised that exposure to natural environments may promote psycho-physiological benefits on people with indications of psychological distress by increasing self-perceived restoration experience and social interactions, being these benefits bigger when the exposure is longer. So, the aims of this study were to investigate: (i) the effects of unconstrained exposure to real natural and urban environments (green, blue, and urban) on psycho-physiological indicators on people with indications of psychological distress; (ii) possible differential effects of 30 minutes and 30+180 minutes of exposure; (iii) possible mechanisms explaining these effects.

## Material and methods

### Participants

All the participants were individuals that had previously participated in a survey of 1000 people in Barcelona city as part of the PHENOTYPE project[11]. As part of the survey, they had answered the Mental Health Inventory (MHI-5) subscale from the SF-36 health questionnaire[22]. The MHI-5 scores had been transformed into a scale from 0 to 100, with higher scores indicating better mental health. So we purposively selected those individuals MHI-5 scored in the lower 50th percentile and who fulfilled the following inclusion criteria: (i) between 18–75 years old, (ii) no smokers, (iii) able to walk for 30 minutes at a self-directed pace, (iv) with no chest, abdomen surgery or heart attack during the last three months, (v) no retinal detachment or eyes surgery or hospitalized for heart problem during the last month, (vi) no under tuberculosis treatment, (vii) no respiratory infection during the last three weeks, (viii) no asthmatic or pregnant, (ix) neither having used inhaler nor taking medication different than usual during the 24 hours previous to any of the data collection days.

Taking into account sample sizes of similarly designed studies[2–4,7,8,12,14,15,17–19,23] and an initial sample size calculation (power of 80% and alpha level of 0.05) we determined that a sample size of 20–50 participants was needed. Hypothesising that people with some psycho-physiological affection would have more marked changes, we pragmatically decided to sample between 25 and 30 participants. Finally, only 26 participants fulfilling the inclusion criteria accepted to participate in the study. The participants that accepted to participate were not different from the ones that either declined to participate or were not possible to contact (S1 Table).

This study was not registered before enrolment of participants started because the National legislation (Royal Decree 223/2004) only requires the registration of those clinical trials with drugs or interventional studies. We considered this study neither of these two study designs, so it was regulated by the Biomedical Research Law 14/2007 and no registration as a clinical trial was needed. However, the study was registered at ClinicalTrials.gov on December 2015 (NCT02624921). And the authors confirm that all ongoing and related trials for this intervention are registered.

Ethical approval (No.2012/4978/l from 18/01/2013) was obtained from the Clinical Research Ethics Committee of the Municipal Health Care (CEIC PS-MAR), Barcelona, Spain. Each participant gave written informed consent and received €150 for participation.

### Design

Participants recruitment and data collection took place in Barcelona Metropolitan Area, in Catalonia, from October 2013 to January 2014. Catalonia is located in the north-east of the Iberian Peninsula and has a typically Mediterranean climate, with mean temperatures of approximately 17°C in October and 7°C in January[24].

Each participant was exposed to all three environments in groups of 2–6 people, always on weekdays, following a case-crossover design. We randomly assigned each data collection day to one exposure environment. Then, we grouped the data collection days so in each group there would be one exposure environment of each type, all the data collection days would be close but at least five days apart. Then, we stuck to these exposure environments groups as much as possible, with the exception of participants schedules not allowing them to participate when assigned. Each participant was assigned to one exposure environment group depending on participant availability. So, the exposure order was randomised as much as possible. No tests were performed during heavy (continuous) rain.

**Fig 1:**
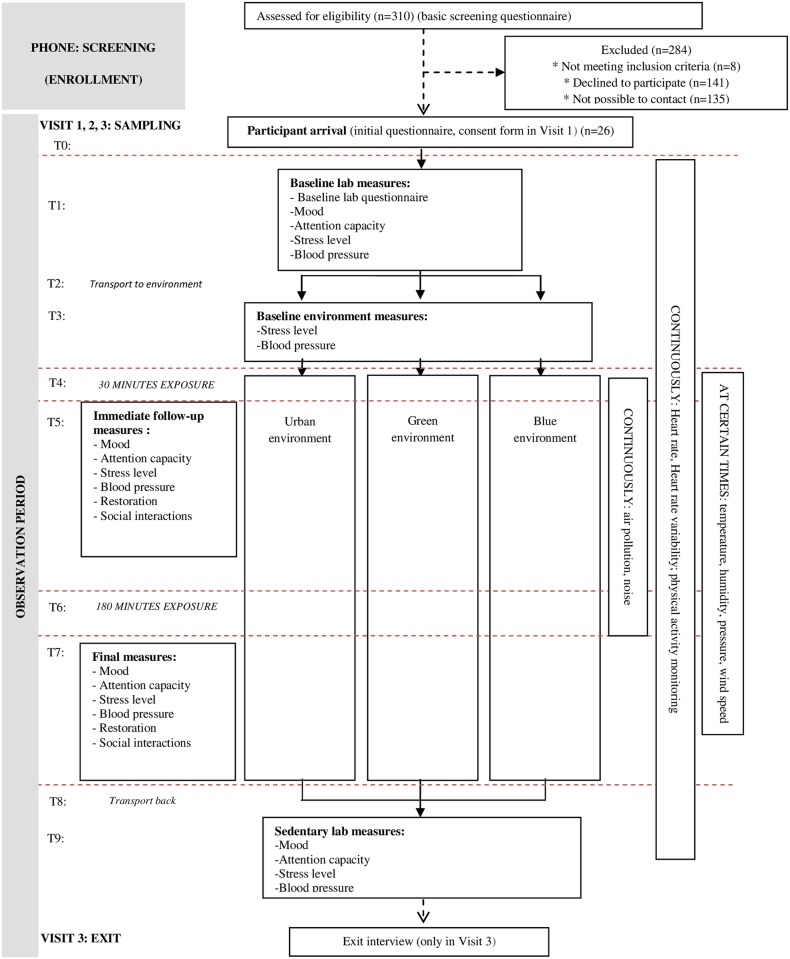
Generic flow graph of sampling days. T0 for Time 0 (participants arrival), T1 for Time 1 (start laboratory measurements), T2 for Time 2 (transport to environment), T3 for Time 3 (initial measurements in the environment), T4 for Time 4 (first exposure, 30 minutes exposure), T5 for Time 5 (measurements after 30 minutes exposure), T6 for Time 6 (second exposure, 180 minutes exposure), T7 for Time 7 (measurements after 180 minutes exposure), T8 for Time 8 (transport to laboratory), T9 for Time 9 (end laboratory measurements).

The protocol was the same for each day of data collection (Fig 1). Participants were instructed to avoid caffeine on the sampling day.

On the first sampling day, participants came to the study lab at 8:30 a.m. to fill in the consent form and an initial questionnaire. The rest of the sampling days, participants came to the lab at 9 a.m. When in the lab (Time 1) measurements were taken. After, we transported participants all together in a passenger van to the exposure environment. The transportation time (Time 2) varied depending on traffic. The one-way transportation time had a median length of 23 (IQR = 21.5, 35) minutes for the green, 25 (24, 25) for the blue, and 19 (18, 21) for the urban exposure environment. Upon arrival at the exposure environment (Time 3), measurements were taken again. After this, we instructed participants to spend 30 minutes in the environment and to come back to the measurements site after this 30 minutes period (Time 4). This exposure was conceived as the short-term exposure, restricted by the time-lag between external events and cortisol changes (20–40 minutes).

After this 30-minute exposure period, we asked participants to sit down again and measurements were taken again (Time 5). After all this, we instructed participants to spend 180 minutes in the environment (Time 6). Also to eat the lunch provided whenever they wanted but always one hour prior to coming back to the measurements site. This exposure was conceived as the mid-term exposure, restricted by the participation hours before their boredom or tireness and by the length of the research team working hours.

After this 180-minute exposure period, we asked participants to sit down again and measurements were taken again (Time 7). After that, we transported participants back to the lab with the same vehicle. The one-way transportation time had a length of 22.5 (IQR = 21.5, 26.5) for the green, 21 (20, 22) for the blue, 17 (17, 18) minutes for the urban exposure environment (Time 8). In the lab (Time 9), we asked participants to sit down again and measurements were taken again. Sampling days usually finished at 6 p.m.

Before the exposure periods participants were instructed to behave as they usually would in that environment, avoiding only swimming, vigorous physical activity and eating (other than the food provided and/or out of schedule). Also, when asked, we indicated that staying as a group during the exposure period was their own decision. It was not possible to blind neither participants nor those assessing the outcomes to the exposure.

### Exposure environments

To enhance ecological validity, our study environments were those widely used by the population of Barcelona, that clearly represented urban, green (NOE without water), or blue environments (NOE with water), and that were similar driving distances from the research centre and parking areas. The environments selected were all in the Barcelona Metropolitan Area including: city, natural park, and beach.

Urban- The Eixample neighborhood in Barcelona is characterized by a grid pattern with long straight streets and octagonal building blocks. It is the most populated neighborhood of Barcelona, with around 16% of Barcelona inhabitants living in it [25]. Our measurements site was located at a square surrounded by busy urban streets (coordinate system WGS 84: 41.383340N, 2.150507 E) (S1 and S2 Figs).

Green- The Collserola Natural Park is a mix forest close to Barcelona city. It is dominated by aleppo pine (*Pinus halepensis*), and oaks (*Quercus ilex* and *Quercus cerrioides*) with dense forest floor. Our measurements site was located close to a picnic area (coordinate system WGS 84: 41.393249 N, 2.091894 E) (S1 and S3 Figs).

Blue—The Castelldefels beach is a river-dominated delta beach with light brown sand of around 100m wide, with small patches of dune herbs. At its edge, there is a promenade seafront of about 12m with no vegetation. At the edge of the promenade there are green areas, streets, and houses. Our measurements site was located in the edge of the promenade (coordinate system WGS 84: 41.265319 N, 1.981937 E) (S1 and S4 Figs).

### Measures

#### Psycho-physiological indicators

**Mood** was assessed using the Spanish short version of the Profile of Mood States (POMS)[26] at time 1, 3, 5, 7, 9. A Total Mood Disturbance (TMD), which indicates negative mood, was obtained from the POMS[27] (Supplemental material, S1 Appendix). Internal consistency reliability of the POMS ranges from 0.84 to 0.95 and test-retest reliability coefficients range from 0.65 to 0.74[17].

**Attention capacity** was assessed with the backwards digit-span task (BDSP) at time 1, 3, 5, 7, 9. Participants heard 14 different sequences from three to nine digits in length (two of each) and repeated them in backward order[28]. The test was completed after two consecutively errors[29]. A total score was obtained which comprised the total number of correct sequences recalled, regardless of digit length. Test-retest reliability coefficients of the BDSP ranges from 0.65 to 0.83[30].

**Stress levels** were assessed using salivary cortisol samples collected using Salivettes(Starstedt, Germany) at time 1, 3, 5, 7, 9. Samples were analyzed in duplicate by Universidad del País Vasco through the standard procedure ELISA (Enzyme-Linked Immuno Sorbent Assay). Salivary cortisol was log-transformed for the analysis. Salivary cortisol is considered to be a reliable and valid parameter of stress reaction in humans[3,12].

**Blood pressure** (systolic and diastolic) measurements were taken using a Digital Blood pressure monitor (Model M10-IT, OMRON Healthcare, UK) at time 1, 3, 5, 7, 9. Measurements were taken in the left arm with a minimum of two measurements taken each time point[31].

**Heart rate** measurements were recorded continuously with a 4-lead electrocardiogram Holter monitor (Model Cardiolight, Gem-Med, Spain) from time 1 to time 9. Raw data were sent to the Holter supplier (Gem-Med) for clinical analysis.

Five-minute periods were selected to represent each time point (time 1, 3, 5, 7, 9). The selection was done considering the fifteen minutes pre-blood pressure measurement (where all the participants were in the same conditions) and also considering the measurements accuracy.

**Autonomous nervous system** (ANS) functioning was assessed with heart rate variability (HRV). HRV measurements were recorded and processed through the same process as the detailed for heart rate. The indicators obtained were: low frequency power (LF; 0.04–0.15Hz), high frequency power (HF; 0.15–0.40Hz), and the ratio between LF and HF (LF:HF). Further, more precise indicators were calculated as coefficients of component variance of LF- and HF (CCV-LF and CCV-HF respectively). They were calculated as the square root of low or high frequency power divided by the mean R-R interval[32,33]. All the frequency-domain indicators were log-transformed for the analysis. Parasympathetic nervous system functioning was derived from HF and CCV-HF. While parasympathetic-sympathetic balance was derived from LF, LF:HF, CCV-LF, and CCV-LF:HF. Absolute reliability, assessed as 95% limits of random variation, is 0.30–3.32 for LF, 0.30–3.36 for HF, 0.28–3.53 for LF/HF. While relative reliability, assessed as the interclass correlation coefficient (95% confidence interval), is 0.79 (0.64–0.88) for LF, 0.86 (0.75–0.92) for HF, and 0.70 (0.50–0.84) for LF/HF [34].

#### Mediators measures

For environmental hazards, we assigned the same value of air pollution/noise to all the participants exposed the same day to the same environment. The value was calculated as the mean value for each day of sampling.

**Air pollution exposure** was assessed measuring black carbon in the air using a Micro-Aethalometer (Model AE51, AethLabs, CA, USA). Measurements were taken every minute from time 4 to 6 in each environment, on each sampling day. Data was corrected applying a smoothing technique that uses the Savitzky—Golay filter.

**Noise exposure** was assessed measuring equivalent A-weighted decibels in the environment using a sonometer (Model SC160, CESVA, Spain). Measurements were taken from time 4 to 6 in each environment, on each sampling day.

**Physical activity** was assessed using CalFit continuously from time 1 to time 9. CalFit is a smartphone-based application configured to collect data on physical activity and geographical location. Total Metabolic Equivalent of Task (MET), total percentage of time in (i) sedentary, (ii) light, (iii) moderate, and (iv) vigorous activities were derived. With the information collected during time 4 we assigned indicators to time 5, and with the information collected during time 6 we assigned indicators to time 7.

**Social interactions** were assessed using self-developed questions at time 5 and 7 (Supplemental Material, S2 Appendix). Total (i) time spent with others and (ii) time spent enjoying talking were derived from these measures.

**Self-perceived restoration experience** was assessed using the six questions of the Restoration outcome scale (ROS)[35] at time 5 and 7. To be consistent with the other parts of the PHENOTYPE project, each question had five response categories (Supplemental material, S3 Appendix). We scored ROS summing up the answers to each question[35] (Supplemental material, S3 Appendix).

Stress levels were assessed as detailed previously. To determine **stress changes**, we calculated the slope between time point of interest (either time 5 or 7) and time 1 (baseline)[36].

#### Exploratory covariates

Information on gender, age, education level, self-perceived general health, self-perceived stress (measured with Perceived stress score that can range between 0 and 40, with 40 indicating a high level of global perceived stress), and chronic illness (yes/no) was collected. Also, on each sampling day, we collected information on self-reported medication intake (yes/no) and waking up time, and measured height and weight. Body Mass Index (BMI) was derived from mean measured height and weight. The difference between waking up time and the time of each measurement was used to calculate elapsed time.

Temperature, relative humidity, pressure and wind speed were measured using a weather station (Model WMR200, Oregon Scientific, OR, USA). Measurements were taken more than once on each sampling day. We assigned the same value of each weather indicator (i.e. temperature/relative humidity/pressure/wind speed) to all the participants exposed the same day to the same environment. The value was calculated as the mean value for each day of sampling.

We also derived a variable to indicate the data collection order through the different environment visits (i.e. sampling order) and one indicating size of exposure group (i.e. group size).

### Statistical analyses

We used multilevel mixed-effects linear regression models with random subject and random outcome baseline levels at time 1 to evaluate the impact of exposure environment on changes in each psycho-physiological indicator and the possible mediators. We also included exposure environment, and time (including time 5 and 7) as fixed effects. In the adjusted models, we also included other covariates as fixed effects, after confirming that multicollinearity was minimal[37]. Mediation and modification were evaluated using the Baron and Kenny approaches[38].

## Results

Table 1 summarizes participant characteristics. Our exposure environments presented very similar physical characteristics, induced similar stress changes and facilitated similar participants' social interactions. However, noise, air pollution, and total physical activity were higher in the urban environment, while restoration was lower in the urban and higher in the blue environment (Table 2).

**Table 1:** Participants characteristics (n = 26).

Characteristic	Frequency (%)^a^
Females	15 (57.69)
Age, median (IQR)	44.32 (26.15)
BMI, median (IQR)	25.94 (7.47)
Education level	
Primary education or less	2 (7.69)
Secondary education	13 (50.00)
University or similar education	11 (42.31)
Self-perceived healthy	18 (69.23)
Mental health index, median (IQR; range)	41.67 (7.39; 26.88, 49.06)
Medication intake, yes	11 (42.31)
Chronic illness, yes	8 (33.33)
Perceived stress scale, median (IQR; range)	15 (9; 4, 29)

^a^ Data are frequency (%) of participants with that characteristic, unless otherwise noted.

SD for standard deviation. IQR for Interquartile range.

**Table 2:** Exposure environments characteristics and descriptive of mediators indicators.

Environment characteristic	Green			Blue			Urban		
	n	median	IQR	n	median	IQR	n	median	IQR
Physical environment									
Temperature (°C)	5	17.43	(15.38, 20.63)	6	20.71	(14.87, 26.43)	5	17.2	(14.9, 24.3)
Relative humidity (%)	5	50.67	(50.33, 54.5)	6	50.33	(40.25, 56.67)	5	42.33	(38, 57)
Wind speed (m/s)	5	1.2	(0.97, 2.1)	6	1.07	(0.38, 1.27)	5	0.23	(0.2, 1.17)
Pressure (mbars)	5	986.67	(975, 999)	6	1017	(1011.67, 1019.25)	5	1012	(1011.33, 1017.75)
Environmental hazards									
Mean value of noise measurements (dB)	5	45.2	(42.41, 59.21)	6	50.91	(48.55, 58.42)	5	65.54	(64.42, 65.72)
Black carbon (µg/m3)	5	1.35	(1.19, 1.67)	6	1.71	(1.37, 2.03)	4	5.47	(4.67, 8.65)
Physical activity									
Total physical activity (METS)	5	2.46	(1.95, 2.79)	5	2.8	(2.41, 3.24)	5	3.07	(2.76, 3.89)
Total time on sedentary activities (%)	5	36	(13.00, 60.29)	5	29	(5.88, 46.67)	5	27.78	(6.67, 40.00)
Total time on light activities (%)	5	30.56	(20.00, 50.00)	5	29.45	(20.00, 40.00)	5	24.17	(18.89, 33.33)
Total time on moderate activities (%)	5	21.67	(9.94, 33.33)	5	30.83	(16.67, 47.06)	5	34.17	(20.00, 56.67)
Total time on vigorous activities (%)	5	0.55	(0.00, 3.33)	5	0	(0.00, 8.89)	5	8.33	(0.00, 16.11)
Social interactions									
Time spent with others (min)	4	30	(29.00, 202.50)	5	30	(30.00, 205.00)	4	30	(20.00, 205.00)
Time spent enjoying talking (min)	4	30	(15.00, 180.00)	4	30	(15.00, 180.00)	4	30	(5.00, 150.00)
Self-perceived restoration experience (n.u.)	5	17.5	(14.00, 21.50)	5	19	(15.00, 21.50)	5	12	(9.00, 15.00)
Stress changes (nmol/L)	5	-3.78	(-9.08, -2.08)	5	-3.95	(-7.34, -1.77)	4	-4.03	(-9.38, -2.23)

METS for Metabolic Equivalent of Task

In our crude models, no statistically significant interaction was found between time and exposure environment for any of the outcomes (S2 Table). No statistically significant association was found between any of the outcomes and the possible covariates BMI, sampling order, or group size (S3 Table). Only small differences were found when using measures at time 1 or time 3 as baseline (S4 and S5 Tables).

In the adjusted models, we found statistically significant lower salivary cortisol when the results after the 210-minute exposure were compared with those after the 30-minute exposure. We found statistically significantly lower TMD, salivary cortisol and HF as a result of being in the green compared with the urban exposure environment, and statistically significantly lower TMD, LF:HF and CCV-LF:HF as a result of being in the blue compared with the urban exposure environment (Table 3). No statistically significant interactions were found by age or gender.

**Table 3:** Associations between exposure environments and psycho-physiological indicators.

Psycho-physiological indicators	n	Green		Blue	
		Coefficient (95% CI)	p-value	Coefficient (95% CI)	p-value
TMD, n.u.	26	-4.78 (-7.77, -1.79)	<0.01	-4.53 (-7.57, -1.49)	<0.01
BDSP, n.u.^a^	26	-0.38 (-0.97, 0.21)	0.20	0.19 (-0.39, 0.77)	0.52
Salivary cortisol, log nmol/L^b^	26	-0.21 (-0.34, -0.08)	<0.01	-0.12 (-0.25, 0.01)	0.06
Blood pressure					
Systolic, mmHg^b^	26	0.36 (-2.58, 3.29)	0.81	-1.30 (-4.21, 1.61)	0.38
Diastolic, mmHg^c^	26	1.42 (-0.34, 3.17)	0.12	-0.40 (-2.14, 1.35)	0.66
Heart rate, beats/min^d^	26	-1.51 (-4.49, 1.47)	0.32	-0.08 (-3.06, 2.91)	0.96
HRV					
HF, log m/s^2^^c^	25	-0.32 (-0.58, -0.06)	0.02	0.01 (-0.24, 0.26)	0.93
LF, log m/s^2^^c^	26	-0.20 (-0.43, 0.02)	0.08	-0.20 (-0.41, 0.02)	0.08
LF:HF, n.u.	22	0.05 (-0.15, 0.25)	0.64	-0.26 (-0.46, -0.06)	0.01
CCV-HF, %^d^	25	-0.03 (-0.31, 0.26)	0.86	0.10 (-0.18, 0.38)	0.47
CCV-LF, %	25	0.10 (-0.20, 0.40)	0.51	-0.01 (-0.31, 0.29)	0.93
CCV-LF:HF, n.u.^b^	25	0.01 (-0.10, 0.13)	0.84	-0.12 (-0.24, -0.01)	0.04

Urban environment as reference environment. TMD for Total Mood Disturbance. BDSP for Backwards Digit-Span task. HRV for Heart Rate Variability. HF for high frequency power. LF for low frequency power. LF:HF for the ratio between LF and HF. CCV-HF for component variance of HF. CCV-LF for component variance of LF. CCV-LF:HF for the component variance of the ratio between LF and HF. Adjusted basic models by participant and baseline measure (at time 1) as random effects, and as fixed effects time, exposure environment, and

^a^temperature and relative humidity,

^b^temperature,

^c^temperature and heart rate,

^d^sampling order.

Estimates and p-values in reference to urban exposure environment.

In the analysis of the possible mediators, only TMD, HF, LF:HF and CCV-LF:HF were explored, as salivary cortisol did not show any association with any of the possible mediators evaluated (S2 Table). For HF, time spent with did not show a statistically significant association when the model was adjusted for other covariates, and for that reason is not reported.

For TMD, both vigorous physical activity and self-perceived restoration experience showed statistically significant associations when introduced separately in the model. For vigorous physical activity, neither green nor blue environments showed statistically significant associations with TMD. For self-perceived restoration experience, green and blue environments showed statistically significant associations with TMD, and its coefficients were lower than in the previous adjusted models.

For LF:HF, air pollution showed a statistically significant association when introduced in the model. When air pollution was introduced, blue environment showed a statistically significant association with LF:HF, but its coefficients being higher than in the previous adjusted model.

For CCV-LF:HF, both light physical activity and air pollution showed statistically significant associations when introduced separately in the model. For light physical activity, blue environment did not show a statistically significant association with CCV-LF:HF. For air pollution, blue environment showed a statistically significant association with CCV-LF:HF, but its coefficient being higher than in the previous adjusted model (Table 4).

**Table 4:** The effects of mediators on the relationship between exposure environment and mood and autonomous nervous system.

Mediators	n (groups)	Green		Blue		Physical activity^a^		Air pollution/Self-perceived restoration experience ^b^	
		Coefficient (95% CI)	p-value	Coefficient (95% CI)	p-value	Coefficient (95% CI)	p-value	Coefficient (95% CI)	p-value
TMD									
Vigorous physical activity	147 (26)	0.33 (-2.49, 3.15)	0.82	1.31 (-1.64, 4.25)	0.39	-1.19 (-1.50, -0.89)	<0.01		
Self-perceived restoration experience	146 (26)	-3.62 (-6.76, -0.47)	0.02	-4.13 (-7.24, -1.03)	0.01			16.26 (0.77, 31.76)	0.04
HRV									
LF:HF									
Air pollution	84 (22)	-0.24 (-0.68, 0.20)	0.29	-0.51 (-0.92, -0.10)	0.02			-0.07 (-0.14, -0.00)	0.04
CCV-LF:HF									
Light physical activity	126 (25)	0.04 (-0.08, 0.17)	0.48	-0.09 (-0.21, 0.03)	0.15	-0.38 (-0.75, -0.01)	0.04		
Air pollution	116 (25)	-0.19 (-0.41, 0.04)	0.10	-0.32 (-0.53, -0.11)	0.00			-0.04 (-0.07, 0.00)	0.05

Urban environment as reference environment. TMD for Total Mood Disturbance. HRV for Heart Rate Variability. LF:HF for the ratio between LF and HF. CCV-LF:HF for the component variance of the ratio between LF and HF.

^a^ refers to either light or vigorous physical activity, according.

^b^ refers to either air pollution, self-perceived restoration experience, according.

Estimates and p-values in reference to urban exposure environment.

## Discussion

This study found that individuals exposed to NOE had better mood, lower stress levels and higher parasympathetic nervous system dominance of the ANS in comparison to those exposed to urban environments. The associations were partially mediated by physical activity, air pollution and self-perceived restoration experience. There was no evidence that the associations between NOE and psycho-physiological indicators were modified by time, gender, or age.

Our findings on mood are in line with previous studies, that showed similar beneficial effects of green environments[3,4,19,21]. Only four studies have reported such short-term effects for blue environments[1,4,16,21]. Our results are in agreement with Sonntag-Öström et al. (2014) and Barton and Pretty (2010) but neither with Marselle et al. (2013) nor Gidlow et al. (2016). The first showed that most mood scales were rated higher when facing a lake (i.e. forest by a lake) compared to when in the city. The second showed that the presence of water in an environment was associated to greater improvements in mood than when only green was present. The Marselle et al. study did not find any association between coastal walks and mood, despite only 6% of their sample performed walks close to the sea, what could deal into low statistical power to detect associations. Similarly, Gidlow et al. did not find any effect of environment on mood changes. Taking into account both our study and the previous research, in unconstrained conditions, both green and blue appear to have a beneficial effect on mood in people with indications of psychological distress.

Moreover, our findings of self-perceived restoration experience and physical activity being partial mediators of the association between exposure environments and mood are novel and have not been explored before.

Our observed lower stress levels in NOE are in line with those from previous studies[3,19]. However, the only two paper studying the short-term effects of green-blue environments on stress levels found no association[8,16]. Our results of beneficial blue environment effects on stress levels almost reached statistical significance. Considering this, and that the only previous evidence involved a different type of NOE (forest with a path leading to a stream, or vegetated footpath besides a canal), no strong conclusions can be drawn.

The unexpected association we found between the green environment and HF is likely to be noise because was not replicated when analyzing CCV-HF. However, the beneficial association we found between the blue environment and LF:HF was replicated when using the more precise indicator CCV-LF:HF. This suggests that the exposure to blue environments leads to the ANS being dominated by the parasympathetic nervous system. These findings are in line with previous studies that showed an increase of parasympathetic activity indicators and/or a decrease of parasympathetic-sympathetic balance indicators when there was an exposure to green or green-blue environments[3,5,7]. Taking into account both our study and the previous research, there are indications that NOE may facilitate physiological relaxation through the promotion of parasympathetic nervous system over the sympathetic nervous system.

Our findings of physical activity and air pollution as possible mediators of the association between exposure environments and ANS are weak and should be interpreted with caution, especially considering that this is novel within the existing literature. The analysis showed that light physical activity increments decrease CCV-LF:HF functioning, as could be expected. While the analysis showed that air pollution increments decrease CCV-LF:HF and LF:HF, contrary to what could be expected, suggesting collider bias due to mediator-outcome confounders not taken into account in the model [39].

Our lack of association between exposure environments and other psycho-physiological indicators is consistent with some previous studies. For example, Gladwell et al. (2012) showed that viewing images from nature did not statistically significantly altered mean heart rate nor blood pressure[7]. While both Lee et al. (2011) and Sonntag-Öström et al. (2014) did find no differences in the blood pressure[4]. However, our lack of associations are contrary to others. For example, Hartig et al. (2003) showed that, when compared to the urban environment, in the natural environment the attention capacity improved and the blood pressure declined more [6]. Lee et al. (2011) found lower heart rate values after the forest exposure[3]. While Sonntag-Öström et al. (2014) found that all natural environments had statistically significantly lower heart rate than in the city, and that the diastolic blood pressure was statistically significantly lower in the forest environments than in the city[4]. And Gidlow et al. found that green and blue environments were associated with more persistent changes in the cognitive function than urban environments[16].

This could be due to our study being the first one in which the exposure involved unconstrained conditions, as Gladwell et al. (2012) used a 5-minutes session of images viewing, while Sonntag-Öström et al. (2014), Tsunetsugu et al. (2013) and Gidlow et al. (2016) used real environments but with highly-controlled exposures[4,5].

Our study found neither indications of association nor modification effects of time, with the exception of stress levels, which can be explained by the typical diurnal pattern of salivary cortisol. Moreover, the lack of interactions between environmental exposure and time would suggest that, considering the outcomes we investigated, no additional benefits are gained from a 30-plus-180-minute compared with a 30-minute exposure. However, it remains unknown if other time points or other psycho-physiological indicators would show statistically significant differences.

This study explored the effects of outdoor environments on psycho-physiological health indicators under unconstrained real conditions, so approximating the participants behaviour to their real-world behaviour, what increased the ecological validity of our study. It is the first to evaluate those effects in a population with indications of psychological distress, separately for green and blue outdoor environments, and including an exploration of the possible mechanisms. Other strengths of our study are the wide use of standardized and validated questionnaires and tests, and objective health measures. Also, the random sampling and the inclusion of male and female participants, which increased our external validity.

Our sample size was modest, which limits our statistical power and, consequently, the strength of our conclusions. However, our study informs about the effect size, so it can be considered in designing future studies. Other limitations of this study are that that we were not able to recruit more people with indications of psychological distress. (e.g. lower 20th percentile), and that we cannot rule out self-selection bias of those with less indications of psychological distress. Despite aiming to explore uncontrolled conditions, we cannot rule out that the study itself influenced participants behaviour and perceptions. For example, our design with exposure in groups of 2–6 people, despite we did not instruct interaction guidelines, could influence our participants behaviour). Looking after time could have effects on participant's health, for example on their stress levels. The design of our study did not allow us to control for important parameters while measuring heart rate variability (i.e., posture or breathing rate), so we cannot rule out measurement error in the indicator derived from HRV measures. Similarly, despite we explored the possible effects of medication intake, we did not explore differential effects of different medications, so we cannot exclude that some residual measurement error is affecting the results on salivary cortisol. Also, despite we randomised the visit order as much as possible and we controlled for it in our analyses, our attention capacity results could still be biased by the learning effects characteristic of most case-crossover studies. Finally, we chose autumn and the beginning of winter because it is the usual period for spending time outdoors on activities that our study design allowed (e.g., walking); in late spring and summer, activities that our study design did not allow are more common (e.g., sunbathing or swimming).

## Conclusions

This paper provides evidence that NOE affect mood and stress levels of people with indications of pyschological distress. Also, that blue environments can affect the parasympathetic-sympathetic balance. This paper also suggests that increased self-perceived restoration experience, increased physical activity, and decreased air pollution are likely to be mediators through which NOE improve psycho-physiological health indicators.

This study extends the existing evidence on the benefits of NOE for people's health. It also suggests NOE potential as a preventive medicine, specifically focusing on people with indications of psychological distress.

Our findings strengthen the existing evidence on the benefits of NOE for people's health. Future studies should try to replicate our findings with uncontrolled experimental study designs with bigger samples than our study. Ideally, the future sample sizes should be enough to stratify the results by age, gender and socioeconomic status. Moreover, it would be interesting if they could evaluate clinically diagnosed people, a greater variety of environments (i.e. several types of green, blue, and urban environments) to evaluate specific characteristics with psycho-physiological effects. Also, if they could investigate several exposure lengths, repeated exposures to the same environments, and how long the psycho-physiological health effects are maintained. The inclusion of individual experiences, beliefs and affinity with nature would also be interesting.

## Supporting information

S1 Table Characteristics comparison between participants and those that declined to participate/not possible to contact. (DOC)

S2 Table Associations between psycho-physiological indicators and possible covariates and mediators. (DOC)

S3 Table Associations between psycho-physiological indicators and possible covariates and mediators. (DOC)

S4 Table Crude associations between exposure environments and psycho-physiological indicators using time 1 as baseline. (DOC)

S5 Table Crude associations between exposure environments and psycho-physiological indicators using time 3 as baseline when possible; if not, time 1. (DOC)

S1 Fig Photos depicting the measurements sites in the urban (left), green (middle), and blue (right) environment settings experienced by participants. (DOC)

S2 Fig Map given to the participants in the urban environment setting. The map depicts the measurements site and some reference points.
(DOC)

S3 Fig Map given to the participants in the green environment setting. The map depicts the measurements site and some reference points.
(DOC)

S4 Fig Map given to the participants in the blue environment setting. The map depicts the measurements site and some reference points.
(DOC)

S1 Appendix Profile of Mood States (POMS) scoring. (DOC)

S2 Appendix Social interactions questions. (DOC)

S3 Appendix Self-perceived restoration experience scoring and questions. (DOC)

S1 CONSORT Checklist (DOC)

S1 Protocol (PDF)
