# Supplementary material for: The effect of randomised exposure to different types of natural outdoor environments compared to exposure to an urban environment on people with indications of psychological distress in Catalonia
Source: PLoS One. 2017 Mar 1;12(3):e0172200. doi: 10.1371/journal.pone.0172200 (PMC5331968; doi:10.1371/journal.pone.0172200)
Supplement: S1 Table — (DOC) [file pone.0172200.s001.doc]

**S1 Table -** Characteristics comparison between participants and those that declined to participate/not possible to contact.

| Characteristic | | Participants (n=26) | Declined to participate or not possible to contact (n=276) | P-value of comparison test between groups |
| --- | --- | --- | --- | --- |
|  |  | Frequency (%) | Frequency (%) |  |
| Gender, female |  | 15 (57.69) | 175 (63.64) | 0.55 |
| Age, median (IQR) | | 44.32 (26.15) | 46.00 (26.00) | 0.14 |
| Self-perceived health, healthy | | 18 (69.23) | 210 (76.36) | 0.42 |
| Smoking status, never smoker | | 15 (57.69) | 188 (68.36) | 0.27 |
| Education | Low | 2 (7.69) | 57 (20.73) | 0.24 |
|  | Medium | 13 (50.00) | 106 (38.55) |  |
|  | High | 11 (42.31) | 112 (40.73) |  |
| Country of birth, Spain | | 6 (23.08) | 72 (26.18) | 0.73 |
| MHI, median (IQR) | | 41.67 (36.74, 44.13) | 41.67 (36.74, 46.59) | 0.98 |
