# Supplementary material for: The effect of randomised exposure to different types of natural outdoor environments compared to exposure to an urban environment on people with indications of psychological distress in Catalonia
Source: PLoS One. 2017 Mar 1;12(3):e0172200. doi: 10.1371/journal.pone.0172200 (PMC5331968; doi:10.1371/journal.pone.0172200)
Supplement: S2 Table — (DOC) [file pone.0172200.s002.doc]

**S2 Table -** Interactions p-values between exposure environment and time. P-value of the chi-2 test comparing the model with and without the interaction term.

|  | |  | |
| --- | --- | --- | --- |
| Psycho-physiological indicators | | | P-value |
|
| TMD | | | 0.86 |
| BDSP | | | 0.92 |
| Salivary cortisol | | | 0.20 |
| Blood pressure | | | |
|  | Systolic | | 0.84 |
|  | Diastolic | | 0.91 |
| Heart rate | | | 0.68 |
| HRV | | | |
|  | HF | | 0.87 |
|  | LF | | 0.73 |
|  | LF:HF | | 0.13 |
|  | CCV-HF | | 0.96 |
|  | CCV-LF | | 0.86 |
|  | CCV-LF:HF | | 0.45 |
|  | |  | |

Urban environment as reference environment. Models adjusted by participant and baseline measure (at time 1 is used as baseline) as random effects.
