# Supplementary material for: The effect of randomised exposure to different types of natural outdoor environments compared to exposure to an urban environment on people with indications of psychological distress in Catalonia
Source: PLoS One. 2017 Mar 1;12(3):e0172200. doi: 10.1371/journal.pone.0172200 (PMC5331968; doi:10.1371/journal.pone.0172200)
Supplement: S3 Table — (DOC) [file pone.0172200.s003.doc]

**S3 Table -** Associations between psycho-physiological indicators and possible covariates and mediators.

| Covariates and mediators | TMD | BDSP | Salivary cortisol | Blood pressure | | Heart rate | HRV | | | | | |
| --- | --- | --- | --- | --- | --- | --- | --- | --- | --- | --- | --- | --- |
|  |  |  |  | Systolic | Diastolic |  | HF | LF | LF:HF | CCV-HF | CCV-LF | CCV-LF:HF |
| Gender | 0.97 | 0.06 | 0.07 | <0.01 | 0.04 | <0.01 | 0.11 | <0.01 | 0.85 | <0.01 | 0.01 | 0.53 |
| Age | 0.80 | 0.04 | 0.17 | 0.48 | 0.07 | 0.02 | <0.01 | 0.01 | 0.97 | <0.01 | 0.06 | 0.43 |
| Body Mass Index | 0.19 | 0.93 | 0.06 | 0.46 | 0.06 | 0.19 | 0.58 | 0.83 | 0.64 | 0.81 | 0.84 | 0.48 |
| Medication intake (yes/no) | 0.51 | 0.37 | 0.14 | 0.49 | 0.93 | 0.62 | 0.09 | 0.03 | 0.47 | 0.25 | 0.60 | 0.21 |
| Sampling order§ | 0.20 | 0.53 | 0.08 | 0.34 | 0.29 | <0.01 | 0.71 | 0.32 | 0.53 | 0.02 | 0.13 | 0.52 |
| Elapsed time | 0.84 | 0.29 | 0.16 | 0.03 | 0.04 | 0.19 | 0.52 | 0.12 | 0.16 | 0.29 | 0.47 | 0.44 |
| Group size | 0.13 | 0.44 | 0.19 | 0.80 | 0.06 | 0.87 | 0.03 | 0.35 | 0.46 | 0.40 | 0.94 | 0.28 |
| Humidity | 0.28 | 0.05 | 0.96 | 0.28 | 0.33 | 0.26 | 0.50 | 0.92 | 0.22 | 0.06 | 0.13 | 0.06 |
| Temperature | 0.80 | 0.05 | 0.03 | <0.01 | 0.00 | 0.20 | <0.01 | 0.01 | 0.09 | 0.83 | 0.52 | 0.03 |
| Pressure | 0.58 | 0.18 | 0.04 | 0.88 | 0.60 | 0.49 | 0.71 | 0.56 | 0.53 | 0.65 | 0.89 | 0.66 |
| Heart rate | 0.83 | 0.91 | 0.15 | 0.68 | 0.03 | - | <0.01 | <0.01 | 0.70 | 0.10 | 0.17 | 0.37 |
| Environmental hazards |  |  |  |  |  |  |  |  |  |  |  |  |
| Air pollution | 0.55 | 0.20 | 0.10 | 0.54 | 0.61 | 0.50 | 0.06 | 0.48 | 0.04 | <0.01 | 0.03 | 0.02 |
| Noise | 0.57 | 0.73 | 0.26 | 0.10 | 0.10 | 0.67 | 0.06 | 0.32 | 0.76 | 0.75 | 0.91 | 0.40 |
| Physical activity |  |  |  |  |  |  |  |  |  |  |  |  |
| Total physical activity | 0.19 | 0.81 | 0.23 | 0.74 | 0.90 | 0.79 | 0.41 | 0.77 | 0.49 | 0.19 | 0.11 | 0.13 |
| Total time on sedentary activities | 0.94 | 0.46 | 0.26 | 0.84 | 0.48 | 0.92 | 0.69 | 0.77 | 0.91 | 0.11 | 0.08 | 0.83 |
| Total time on light activities | 0.28 | 0.21 | 0.26 | 0.98 | 0.86 | 0.88 | 0.46 | 0.65 | 0.26 | 0.71 | 0.85 | 0.05 |
| Total time on moderate activities | 0.94 | 0.48 | 0.09 | 0.82 | 0.44 | 0.92 | 0.89 | 0.29 | 0.15 | 0.47 | 0.12 | 0.06 |
| Total time on vigorous activities | 0.04 | 0.75 | 0.98 | 0.96 | 0.46 | 0.93 | 0.57 | 0.29 | 0.50 | 0.69 | 0.84 | 0.83 |
| Self-perceived restoration experience | <0.01 | <0.01 | 0.55 | 0.29 | 0.25 | 0.43 | 0.52 | 0.41 | 0.52 | 0.17 | 0.38 | 0.53 |
| Stress changes | 0.29 | 0.82 | - | 0.78 | 0.92 | 0.16 | 0.15 | 0.41 | 0.78 | 0.99 | 0.81 | 0.66 |
| Social interactions |  |  |  |  |  |  |  |  |  |  |  |  |
| Time spent with somebody else | 0.27 | 0.75 | 0.81 | 0.64 | 0.87 | 0.08 | 0.02 | 0.11 | 0.17 | 0.22 | 0.41 | 0.18 |
| Time spent enjoying talking | 0.39 | 0.90 | 0.47 | 0.53 | 0.87 | 0.45 | 0.14 | 0.47 | 0.40 | 0.02 | 0.07 | 0.18 |

Adjusted models by participant and baseline measure (at time 1) as random effects, and as fixed effects time and exposure environment. P-values of each covariate/mediator in the model reported with the exception of the variable "sampling order"§ where the likelihood ratio test p-value of including the "sampling order" variable in the model or not is reported.
