# Supplementary material for: The effect of randomised exposure to different types of natural outdoor environments compared to exposure to an urban environment on people with indications of psychological distress in Catalonia
Source: PLoS One. 2017 Mar 1;12(3):e0172200. doi: 10.1371/journal.pone.0172200 (PMC5331968; doi:10.1371/journal.pone.0172200)
Supplement: S4 Table — (DOC) [file pone.0172200.s004.doc]

**S4 Table -** Crude associations between exposure environments and psycho-physiological indicators using time 1 as baseline.

|  | |  | | | | | |
| --- | --- | --- | --- | --- | --- | --- | --- |
| Psycho-physiological indicators | | | n (group) | Green | | Blue | |
|  |  |  |  | Coefficient (95% CI) | p-value | Coefficient (95% CI) | p-value |
| TMD | | | 147 (26) | -4.78 (-7.77, -1.79) | <0.01 | -4.53 (-7.57, -1.49) | <0.01 |
| BDSP | | | 138 (26) | -0.45 (-1.03, 0.12) | 0.12 | 0.00 (-0.58, 0.59) | 0.99 |
| Salivary cortisol | | | 149 (26) | -0.18 (-0.30, 0.05) | 0.01 | -0.14 (-0.27, -0.02) | 0.03 |
| Blood pressure | | | |  |  |  |  |
|  | Systolic | | 154 (26) | 1.63 (-1.37, 4.62) | 0.29 | -2.38 (-5.37, 0.62) | 0.12 |
|  | Diastolic | | 154 (26) | 1.99 (0.13, 3.85) | 0.04 | -1.13 (-2.99, 0.72) | 0.23 |
| Heart rate | | | 154 (26) | -1.56 (-4.55, 1.42) | 0.31 | 0.14 (-2.84, 3.12) | 0.93 |
| HRV | | | |  |  |  |  |
|  | HF | | 146 (25) | -0.11 (-0.40, 0.18) | 0.47 | -0.06 (-0.35, 0.24) | 0.71 |
|  | LF | | 149 (26) | -0.08 (-0.33, 0.17) | 0.54 | -0.24 (-0.49, 0.01) | 0.06 |
|  | LF:HF | | 94 (22) | 0.05 (-0.15, 0.25) | 0.64 | -0.26 (-0.46, -0.06) | 0.01 |
|  | CCV-HF | | 128 (25) | 0.11 (-0.17, 0.39) | 0.45 | 0.08 (-0.20, 0.37) | 0.57 |
|  | CCV-LF | | 128 (25) | 0.10 (-0.20, 0.40) | 0.51 | -0.01 (-0.31, 0.29) | 0.93 |
|  | CCV-LF:HF | | 126 (25) | -0.03 (-0.14, 0.09) | 0.64 | -0.10 (-0.21, 0.02) | 0.11 |
|  | |  | | | | | |

Urban environment as reference environment. Models adjusted by participant and baseline measure (at time 1 is used as baseline) as random effects, time and exposure environment as fixed effects.
