# Supplementary material for: The effect of randomised exposure to different types of natural outdoor environments compared to exposure to an urban environment on people with indications of psychological distress in Catalonia
Source: PLoS One. 2017 Mar 1;12(3):e0172200. doi: 10.1371/journal.pone.0172200 (PMC5331968; doi:10.1371/journal.pone.0172200)
Supplement: S5 Table — (DOC) [file pone.0172200.s005.doc]

**S5 Table -** Crude associations between exposure environments and psycho-physiological indicators using time 3 as baseline when possible; if not, time 1.

| Psycho-physiological indicators | | | | n (group) |  | Green | | Blue | |
| --- | --- | --- | --- | --- | --- | --- | --- | --- | --- |
|  |  |  |  |  |  | Coefficient (95% CI) | p-value | Coefficient (95% CI) | p-value |
| TMD | | | | 147 (26) |  | -4.78 (-7.77, -1.79) | <0.01 | -4.53 (-7.57, -1.49) | <0.01 |
| BDSP | | | | 138 (26) |  | -0.45 (-1.03, 0.12) | 0.12 | 0.00 (-0.58, 0.59) | 0.99 |
| Salivary cortisol | | | | 152 (26) |  | -0.21 (-0.35, -0.08) | <0.01 | -0.18 (-0.32, -0.05) | 0.01 |
| Blood pressure | | | |  |  |  |  |  |  |
|  | | Systolic | | 154 (26) |  | 1.53 (-1.24, 4.31) | 0.28 | -1.50 (-4.28, 1.29) | 0.29 |
|  | | Diastolic | | 154 (26) |  | 1.81 (0.00, 3.64) | 0.05 | -0.77 (-2.60, 1.06) | 0.41 |
| Heart rate | | | | 154 (26) |  | -1.62 (-4.62, 1.37) | 0.29 | 0.10 (-2.90, 3.09) | 0.95 |
| HRV | | | | |  |  |  |  |  |
|  | HF | | | 154 (26) |  | -0.20 (-0.47, 0.07) | 0.15 | -0.13 (-0.41, 0.14) | 0.34 |
|  | LF | | | 151 (26) |  | -0.08 (-0.33, 0.16) | 0.52 | -0.24 (-0.48, 0.01) | 0.06 |
|  | LF:HF | | | 105 (25) |  | 0.12 (-0.08, 0.32) | 0.23 | -0.05 (-0.24, 0.15) | 0.64 |
|  | CCV-HF | | | 134 (26) |  | 0.09 (-0.18, 0.36) | 0.52 | 0.02 (-0.25, 0.29) | 0.88 |
|  | CCV-LF | | | 134 (26) |  | 0.14 (-0.14, 0.42) | 0.33 | -0.08 (-0.36, 0.20) | 0.58 |
|  | CCV-LF:HF | | | 130 (26) |  | 0.04 (-0.07, 0.16) | 0.45 | -0.10 (-0.21, 0.02) | 0.10 |
|  | | |  | | | | | |  |

Urban environment as reference environment. Models adjusted by participant and baseline measure (at time 3 with the exception of cognitive and mood, where time 1 is used as baseline) as random effects, time and exposure environment as fixed effects.
