# Supplementary material for: The effect of randomised exposure to different types of natural outdoor environments compared to exposure to an urban environment on people with indications of psychological distress in Catalonia
Source: PLoS One. 2017 Mar 1;12(3):e0172200. doi: 10.1371/journal.pone.0172200 (PMC5331968; doi:10.1371/journal.pone.0172200)

**S1 Fig. -** Photos depicting the measurements sites in the urban (left), green (middle), and blue (right) environment settings experienced by participants.
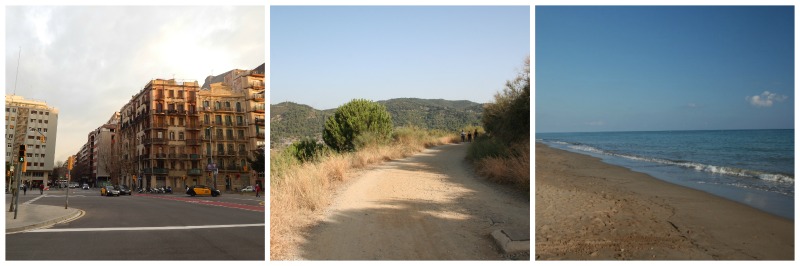

Supplement: S1 Fig — (DOC) [file pone.0172200.s006.doc]
