# Supplementary material for: The effect of randomised exposure to different types of natural outdoor environments compared to exposure to an urban environment on people with indications of psychological distress in Catalonia
Source: PLoS One. 2017 Mar 1;12(3):e0172200. doi: 10.1371/journal.pone.0172200 (PMC5331968; doi:10.1371/journal.pone.0172200)

**S2 Fig. -** Map given to the participants in the urban environment setting. The map depicts the measurements site and some reference points.


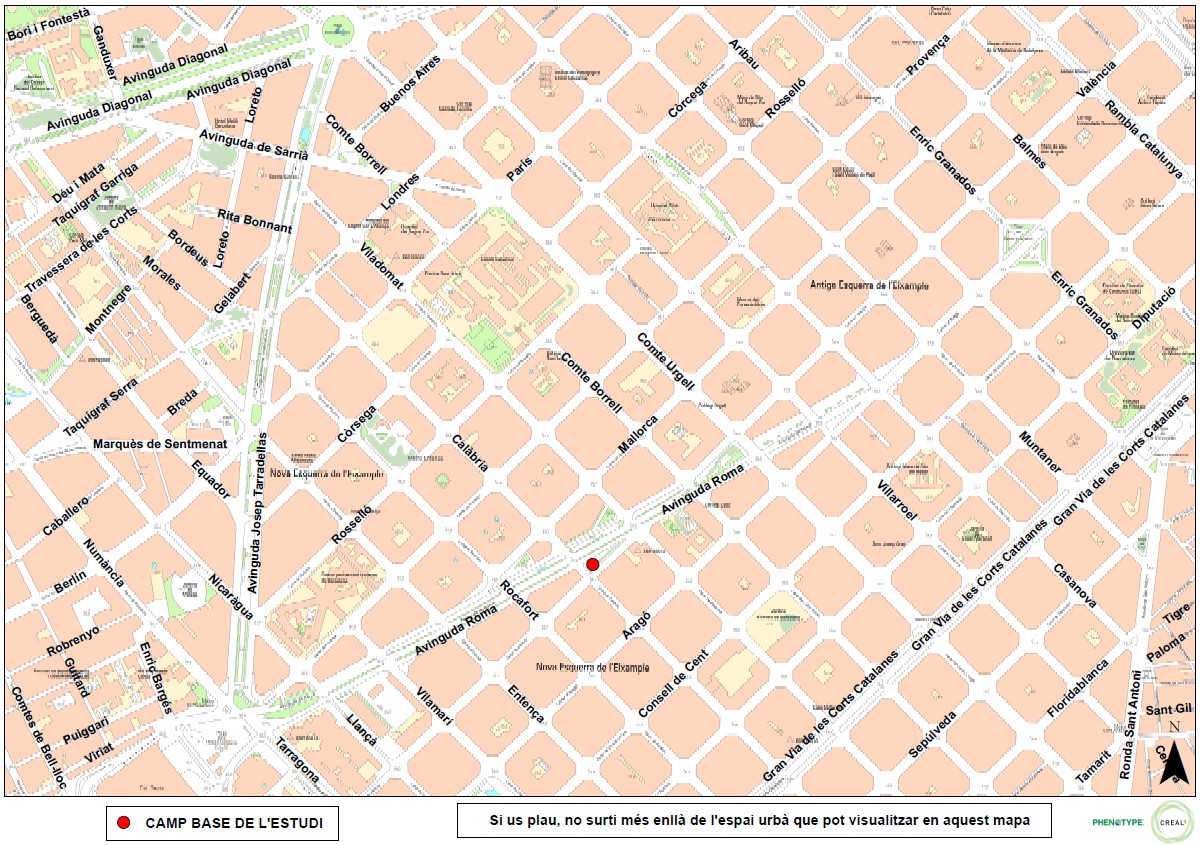

Supplement: S2 Fig — The map depicts the measurements site and some reference points. (DOC) [file pone.0172200.s007.doc]
