# Supplementary material for: The effect of randomised exposure to different types of natural outdoor environments compared to exposure to an urban environment on people with indications of psychological distress in Catalonia
Source: PLoS One. 2017 Mar 1;12(3):e0172200. doi: 10.1371/journal.pone.0172200 (PMC5331968; doi:10.1371/journal.pone.0172200)
Supplement: S1 Appendix — (DOC) [file pone.0172200.s010.doc]

**S1 Appendix - Profile of Mood States (POMS) scoring**

Below is a list of items that describe how people feel. Please, read each one carefully. For each item please **circle the number** that best describe **HOW YOU FEEL RIGHT NOW**.

|  | **Not at all** | **A little** | **Moderately** | **Quite a bit** | **Extremely** |
| --- | --- | --- | --- | --- | --- |
| Intranquilo | 0 | 1 | 2 | 3 | 4 |
| Energético | 0 | 1 | 2 | 3 | 4 |
| Desamparado | 0 | 1 | 2 | 3 | 4 |
| Furioso | 0 | 1 | 2 | 3 | 4 |
| Sin fuerzas | 0 | 1 | 2 | 3 | 4 |
| Deprimido | 0 | 1 | 2 | 3 | 4 |
| Lleno de energía | 0 | 1 | 2 | 3 | 4 |
| Inquieto | 0 | 1 | 2 | 3 | 4 |
| Molesto | 0 | 1 | 2 | 3 | 4 |
| Agotado | 0 | 1 | 2 | 3 | 4 |
| Agitado | 0 | 1 | 2 | 3 | 4 |
| Luchador | 0 | 1 | 2 | 3 | 4 |
| Desdichado | 0 | 1 | 2 | 3 | 4 |
| Irritable | 0 | 1 | 2 | 3 | 4 |
| Cansado | 0 | 1 | 2 | 3 | 4 |
| Amargado | 0 | 1 | 2 | 3 | 4 |
| Animado | 0 | 1 | 2 | 3 | 4 |
| Nervioso | 0 | 1 | 2 | 3 | 4 |
| Enfadado | 0 | 1 | 2 | 3 | 4 |
| Exhausto | 0 | 1 | 2 | 3 | 4 |
| Tenso | 0 | 1 | 2 | 3 | 4 |
| Vigoroso | 0 | 1 | 2 | 3 | 4 |
| Triste | 0 | 1 | 2 | 3 | 4 |
| Enojado | 0 | 1 | 2 | 3 | 4 |
| Fatigado | 0 | 1 | 2 | 3 | 4 |
| Infeliz | 0 | 1 | 2 | 3 | 4 |
| Activo | 0 | 1 | 2 | 3 | 4 |
| Relajado | 4 | 3 | 2 | 1 | 0 |
| De mal genio | 0 | 1 | 2 | 3 | 4 |
