# Supplementary material for: The effect of randomised exposure to different types of natural outdoor environments compared to exposure to an urban environment on people with indications of psychological distress in Catalonia
Source: PLoS One. 2017 Mar 1;12(3):e0172200. doi: 10.1371/journal.pone.0172200 (PMC5331968; doi:10.1371/journal.pone.0172200)
Supplement: S2 Appendix — (DOC) [file pone.0172200.s011.doc]

**S2 Appendix -** **Social interactions questions**

| Since you arrived to this environment, how much time have you spent alone? (approximately) | ___h | __min |
| --- | --- | --- |
| Since you arrived to this environment, how much time have you been with other people? (not including the research team) (approximately) | ___h | __min |
| Since you arrived to this environment, how much time have you been talking with other people? (not including the research team) (approximately) | ___h | __min |
| Since you arrived to this environment, how much time have you been enjoying talking with other people? (not including the research team) (approximately) | ___h | __min |
