# Supplementary material for: The effect of randomised exposure to different types of natural outdoor environments compared to exposure to an urban environment on people with indications of psychological distress in Catalonia
Source: PLoS One. 2017 Mar 1;12(3):e0172200. doi: 10.1371/journal.pone.0172200 (PMC5331968; doi:10.1371/journal.pone.0172200)
Supplement: S3 Appendix — (DOC) [file pone.0172200.s012.doc]

**S3 Appendix - Self-perceived restoration experience**  **scoring and questions**

Please read each statement carefully, then ask yourself, “How much does this statement apply to my experience here?”

To indicate your answer, circle one of the numbers on the scale.

|  | **Not at all** | **A little** | **Somewhat** | **Much** | **Very Much** |
| --- | --- | --- | --- | --- | --- |
| **1.** I feel calmer | 0 | 1 | 2 | 3 | 4 |
| **2.** After visiting this place I feel restored and relaxed | 0 | 1 | 2 | 3 | 4 |
| **3.** I have new enthusiasm and energy for my everyday routines from here | 0 | 1 | 2 | 3 | 4 |
| **4.** My concentration and alertness clearly increased | 0 | 1 | 2 | 3 | 4 |
| **5.** I forgot everyday worries here | 0 | 1 | 2 | 3 | 4 |
| **6.** Visiting here cleared and clarified my thoughts | 0 | 1 | 2 | 3 | 4 |
