# Supplementary material for: The effect of randomised exposure to different types of natural outdoor environments compared to exposure to an urban environment on people with indications of psychological distress in Catalonia
Source: PLoS One. 2017 Mar 1;12(3):e0172200. doi: 10.1371/journal.pone.0172200 (PMC5331968; doi:10.1371/journal.pone.0172200)
Supplement: S1 Protocol — (PDF) [file pone.0172200.s014.pdf]

PHENOTYPE
Health from outside in

Workpackage 4 Study Protocol - Spain

For Ethical purpose

- Therapeutic studies -

PHENOTYPE, Positive Health Effects of the Natural Outdoor environment in Typical Populations in different regions in Europe

FP7-ENV-2011-Grant Agreement number: 282996

SEVENTH FRAMEWORK PROGRAMME

WP no	4
WP title	Therapeutic Studies

CONTENTS

Contents ... 3
1. INTRODUCTION ... 4
1.1. AFFECTIVE RESPONSES ... 4
1.2. ATTENTION RESTORATION ... 5
1.3. PHYSIOLOGICAL EFFECTS ... 5
1.4. TEMPORAL PATTERNING OF EFFECTS ... 5
1.5. EXTENSION OF THE LITERATURE ... 6
PHYSIOLOGICAL RESPONSES ... 6
INSTORATIVE EFFECTS ... 7
SUSTAINED EFFECTS ... 7
METHODOLOGICAL ADVANCES ... 8
2. OBJECTIVES ... 9
3. METHODS ... 10
3.1. DESIGN ... 10
3.2. STUDY POPULATION ... 12
3.3. DATA COLLECTION ... 12
HEALTH OUTCOMES ... 13
EXPOSURE MONITORING ... 14
4. DATA MANAGEMENT ... 15
5. DATA ANALYSES ... 15
6. POTENTIAL RISKS ... 15
6.1. INSURANCE ... 15
7. FINANCING ... 15
8. PAYMENT OF PARTICIPANTS ... 15
9. REFERENCES ... 16
APPENDIX 1: INFORMATION FOR PARTICIPANTS ... 19
APPENDIX 2: CONSENT FORMS ... 21
APPENDIX 3: BASELINE PROFILING QUESTIONNAIRE ... 22
APPENDIX 4: COGNITIVE FUNCTIONING (BACKWARDS DIGIT SPAN TASK) ... 36
APPENDIX 5: RESTORATION OUTCOMES SCALE ... 38
APPENDIX 6: SOCIAL INTERACTIONS QUESTIONNAIRE ... 39

1. INTRODUCTION

A growing body of evidence has indicated a positive relationship between natural environment exposure and human health and well-being (Maas et al., 2006; Mitchell & Popham, 2008). Investigating the underlying mechanisms mediating this relationship is complex. Mechanisms that have been implicated, and which are under investigation within WP2 (large community survey) and WP3 (epidemiological data from large cohort studies), include: stress reduction; restorative function; behaviour (i.e. physical activity); social interaction; and reduction of environmental hazards (e.g., noise, air pollution). WP4 will take an experimental approach to measure affective, cognitive and physiological responses to natural environment exposure to further explore these mechanisms. Data will be collected from burn out people to improve our understanding of the therapeutic effects of natural environments.

Two theories have dominated explanations for the beneficial effects of natural environments; the attention restoration theory (ART) proposed by Kaplan, & Kaplan (1989) (Kaplan, 1995) and the stress reduction theory (SRT) proposed by Ulrich, (1983) (Ulrich et al., 1991).

The ART proposes that nature provides the particular environmental stimuli to allow restoration from directed attention fatigue. Directed attention is described as a critical resource for executive functioning and is susceptible to depletion (Kaplan & Berman, 2010). Restoration from mental fatigue can proceed when four factors characterise the person-environment interaction; psychological and geographical distancing from aspects of one's usual environments, routines and situations (being away), immersion in a coherent physical or conceptual environment that is of sufficient scope to sustain exploration (extent), effortless attention as drawn by objects in the environment or engaged in the process of making sense of the environment (fascination), and a good match between personal inclinations and purposes, environmental supports for intended activities, and environmental demands for action (compatibility) (Kaplan, 1995) . People's readiness to respond positively to nature is evidenced in that attention is easily and almost effortlessly held by natural scenes; referred to as 'soft fascination' (Kaplan & Kaplan, 1989). When nature captures attention, executive systems that regulate directed attention get to rest, pessimistic thoughts are blocked, and negative emotions are replaced by positive ones (Parsons, 1991).

In comparison, the stress reduction theory (SRT) (Ulrich, 1983) posits that environments promote recovery from stress and help to moderate and diminish states of arousal and negative thoughts, through psycho-physiological pathways (Hartig et al., 2003). This positive response to natural environments is typically explained from an evolutionary perspective and reflects a response to habitable settings that were favourable to well-being and survival for pre-modern people (Kellert & Wilson, 1993).

A number of state of the art reviews have been conducted to synthesise the evidence in this area and, in general, this research has shown more positive affective, cognitive, and physiological responses to natural settings than to urban settings (Barton & Pretty, 2010; Bowler et al., 2010; Bratman et al., 2012; Thompson Coon et al., 2011). The findings of field studies are also supported by laboratory experiments in which affective, cognitive, and physiological responses to viewing visual simulations of natural and urban environments have been assessed (Berto, 2005; Ulrich et al., 1991, van den Berg et al., 2003). In sum, there is convergent evidence that contact with real, or simulated, natural environments can provide restoration from stress and mental fatigue. This convergent evidence is now considered in more detail.

1.1. AFFECTIVE RESPONSES

There is consistent evidence that exposure to natural environments improve psychological wellbeing. In a meta-analysis (Barton & Pretty, 2010) assessed the best dose of exposure to green exercise required to improve self esteem and mood. Mental health outcomes were assessed for several types of green exercise (e.g., walking, horse riding, sailing, gardening) in different habitats (e.g., woodland, forests, watersides, urban green areas, farmland natural habitats). Exercise in all natural

environments resulted in significant improvements in both self-esteem and mood. However, habitats with open water produced a significantly larger degree of improvement. Improvements in self-esteem and mood were observed after acute exposure/activity (during the first 5 minutes) with diminishing, but still positive returns, thereafter. The reviewed studies did not, however, include comparator urban or built environments. In a recent systematic review physical activity in natural environments was reported to be associated with decreased feelings of tension, confusion, anger and depression, while exhibiting greater feelings of revitalisation (Thompson Coon et al., 2011). Similar positive effects on mood for walking or running in natural environments were reported by Bowler et al, 2010(Bowler et al., 2010). Outdoor experiences are also rated as more restorative (Hug et al., 2009) and more effective in improving mood and vitality (Ryan et al., 2010). In comparison, indoor activity was associated with increased frustration, anxiety, anger and sadness (Teas et al., 2007). In sum there is consistent evidence that engaging with natural environments is associated with positive changes in self-reported affect and psychological state.

1.2. ATTENTION RESTORATION

There have been multiple empirical studies that have supported the ART, and provided evidence that interaction with natural environments facilitates restoration of directed attention (Berman et al., 2008; Berto, 2005; Staats et al., 2003; Tennessen & Cimprich, 1996; van den Berg et al., 2003). To illustrate, Berman et al. (2008) demonstrated that walking for an hour in a park resulted in significantly better improvements in tests of directed attention than walking for an hour in an urban area (as measured by a Backward Digit Span Test). Directly interacting with nature is not necessary to achieve the attention restoration effect. For example, viewing a natural environment can be enough to improve directed attention abilities, with students who have a view of the natural environment from their dormitory windows scoring significantly higher on several measures of attention than students who had any other type of view (mostly natural, mostly urban, and all urban) from their window (Tennessen & Cimprich, 1996). Restoration effects have also been observed in a laboratory in less than 10 minutes, but viewing natural environments high on perceived fascination was needed to restore ability to focus attention after a prolonged and intense mental effort (Berto, 2005; Hartig et al., 2003).

1.3. PHYSIOLOGICAL EFFECTS

Fewer studies have focused on physiological effects of engagement with natural environments compared with self-reported changes in psychological state. While there is some evidence from well controlled studies (Hartig et al., 2003) that engagement with a natural environment can lower blood pressure, in general the evidence relating to physiological responses following engagement with a natural environment has proved inconsistent (Bowler et al., 2010). Restorative effects of viewing natural environments have, however, been shown with relative consistency in laboratory studies that have investigated improvements following a stressor; changes from baseline in 'unstressed' individuals have been less studied. For example, when participants were exposed to a stressor (elevating heart rate (HR) and blood pressure (BP)) prior to viewing videos of different environments, natural views were regarded as more 'restorative' because they elicited more rapid returns to baseline HR (Laumann, Gärling, & Stormark, 2003; Ulrich et al., 1991) and BP (Chang, Hammitt, Chen, Machnik, & Su, 2008). When treadmill exercise has been incorporated into laboratory-based simulated environment exposure, BP was lower after a 5-min recovery period after viewing slides depicting natural scenes when compared with slides depicting built environments (Pretty, Peacock, Sellens, & Griffin, 2005). Despite some studies demonstrating physiological responses to natural environments there is a need for further field-based physiological measures in this area.

1.4. TEMPORAL PATTERNING OF EFFECTS

Consideration of the pattern of affective, cognitive and physiological responses to engagement with the natural environment may help to elucidate the relative merits of ART and SRT as potential explanations for the beneficial effects of engagement with the natural environment. To explain, research has demonstrated differential temporal patterns in affective, cognitive and physiological

responses to natural and urban environment exposure with some effects appearing after others have dissipated. Physiological changes have occurred within a few minutes of initial exposure to a natural environment in both laboratory (Ulrich et al., 1991) and actual natural environments (Hartig et al., 2003). Similarly acute responses are observed for changes in mood and self-esteem (Barton & Pretty, 2010). In short, psycho-physiological responses to natural environments, indicating a reduction in stress levels as suggested by SRT, appear quickly and support the notion of a 'hard-wired' positive evolutionary response to natural environments. In contrast, changes in performance on cognitive tasks did *not* consistently emerge following 15-20 minutes of environment exposure (via videotape) (Laumann et al., 2003), but did emerge after 50 minutes engagement in a natural environment (Berman, et al., 2008; Hartig, et al., 2003). The temporal patterns observed suggest the possibility of different causal pathways operating for positive impacts for stress-reduction (SRT) and attention replenishment (ART) of natural environment exposure (Bratman et al., 2012). This can be explored through field-based experiments that incorporate longer term follow up and repeated exposures.

1.5. EXTENSION OF THE LITERATURE

Based on the evidence to date there are a number of areas that warrant further study and will be explored through WP4. The section below will outline how the literature can be extended through the use of more robust physiological measures, a focus on instorative effects, consideration of the sustained effects of exposure to a natural environment, adopting a predominantly field-based approach to data collection; greater attention to methodological rigour that can minimise potential confounds in field based studies by controlling for, or testing the effects of: physical activity, social interaction, attitudes to natural environment and the selection of the environment.

PHYSIOLOGICAL RESPONSES

We aim to extend the current physiological evidence base in studies of healthy and clinical populations, using two robust measures of well-being (cortisol and Heart Rate Variability (HRV)), and an experimental design that enable enquiry beyond the immediate exposure effects.

Heart Rate Variability (HRV)

The ability to regulate our emotions is crucial across a range of settings (Gross, 1998). HRV is a measure of autonomic flexibility, that is the interplay between sympathetic and parasympathetic influences on heart rate, and is proposed to represent the capacity for regulated emotional response (Appelhans & Luecken, 2006). Greater HRV is associated with more positive emotional response to stressors (Bornas et al., 2005) and performance on stressful tasks (Hansen, Johnsen, & Thayer, 2003). A comparison of viewing 18 images of natural versus urban environments over five minutes (per condition) showed greater HRV in the natural environment condition (Gladwell et al., 2012). However, to date little work has explored HRV changes in response to actual engagement with the natural environment. Existing evidence is somewhat limited by methodological limitations in field-based experiments in which changes from baseline levels are not outlined. This study seeks to build on the emerging work in this area to explore if similar effects are observed following actual engagement in a natural environment. Because HRV is a potential (and objective) indicator of how individuals respond and perform under stress it is an excellent indicator of the potential preventative benefits of engagement with the natural environment; i.e. increased HRV in response to the natural environment could indicate a role in preventing subsequent debilitating responses to stress. The present research will contribute to the literature by demonstrating the impact of engagement with a natural environment on HRV.

Cortisol

A consistent body of research has demonstrated that both physical and psychological stressors can increase cortisol levels, through the activation of the hypothalmic-pituitary-adrenocortical (HPA) axis (Dickerson & Kemeny, 2004; Hellhammer, Wüst, & Kudielka, 2009). Cortisol plays an important role in mobilising energy resources and regulating a number of physiological symptoms. However,

heightened HPA activity is also associated with a number of negative effects such as, depressive symptoms, suppression of the immune system and development or progression of chronic diseases such as diabetes or hypertension (Dickerson & Kemeny, 2004). Despite cortisol being a robust and frequently used measure of psychological stress, with meaningful health consequences, it has rarely been measured in response to engagement with a natural environment. An exploratory study of Scottish adults reported a positive correlation between the diurnal decline in cortisol across the day and percentage of green space in the neighbourhood environment, independent of socio-demographic variables. This is an important first step towards using robust physiological stress measures in population studies. But cause-effect regarding cortisol and actual *engagement* with nature (rather than 'proximity to' or 'neighbourhood greenness') has not yet been determined.

When favourable cortisol response to natural environments has been reported in field settings, there have either been methodological limitations, such a failure to account for the diurnal pattern of cortisol, or the focus has been on recovery following a stressor (van den Berg & Custers, 2011). There remains a lack of evidence that engagement with a natural environment can alter levels of cortisol from those typically experienced in day- to-day life. Because cortisol is a robust indicator of response to stress and is associated with negative health consequences it is an excellent indicator of both the potential preventative and therapeutic benefits of engagement with the natural environment. The research in WP4 will contribute to the literature by demonstrating the impact of engagement with a natural environment on cortisol.

INSTORATIVE EFFECTS

There is increasing evidence that exposure to nature / natural environments may not only have restorative effects, but also instorative effects in individuals who are not stressed or fatigued (Hartig, 2007). Recent studies among healthy, unstressed individuals have shown that exposure to nature may improve mood states and an individual's ability to reflect (Mayer et al., 2008) and increase self-reported 'vitality' (Ryan et al., 2010). In short, engagement with natural environments may have wider public health benefits, than facilitating recovery in individuals experiencing elevated levels of stress. This approach dovetails with the growing 'positive psychology movement' which focuses on understanding and enhancing positive aspects of life, rather than simply focusing on reducing negative aspects (Seligman & Csikszentmihalyi, 2000). That is, to understand how engagement with the natural environment can bring about positive change in terms of enhancing well-being, rather than only reducing negative aspects. To this end, in addition to a range of affective, cognitive and physiological measures, data on eudemonic and hedonic aspects of well-being will be collected.

SUSTAINED EFFECTS

As discussed previously, temporal patterns in responses to natural environment suggest the possibility of different causal pathways operating for positive impacts for SRT and ART (Bratman et al., 2012). This study will incorporate measurement of responses that are immediate (e.g., 30-minutes post exposure) and medium-term (e.g., 30-minutes following end of exposure). Moreover, this study will incorporate measurements after short-exposure (30 minutes of exposure) and after long-exposure (270 minutes of exposure). This should provide further understanding of whether any immediate changes are sustained and if the amount of exposure changes the outcomes. In short, it provides an experimental test of the affective, physiological and cognitive consequences of how people typically engage with natural environments, particularly in urban environments. That is, long-term exposures on single (e.g., a daily trip to the park once a month), and short-term (e.g., daily trip to the same park every lunchtime) occasions. This research will contribute to the literature by exploring the sustained effects of short-term and long-term engagement with the natural environment that hitherto have not been considered.

METHODOLOGICAL ADVANCES

Field versus Laboratory Experiments

Different approaches to experimental exposure have been used in research to date, broadly grouped into use of images (including videos), views through windows, and being present in and being physically active in environments (Bowler et al., 2010; Bratman et al., 2012).

Often researchers have used laboratory settings to control the specific type of environment exposures through showing participants images or videos of various scenes, often following some kind of image classification process (Laumann et al., 2001; Pretty et al., 2007; White et al., 2010). The obvious advantage is the internal validity through controlling for potential confounders and the ability for individual participants to experience a large number of different environments. The ecological validity, however, is limited as engaging with an environment involves more than visual stimuli; for example, smells, sounds and dynamic nature of environments cannot be experienced. The potential effects of engaging in with a natural environment may, therefore, be underestimated.

There are numerous examples of field-based experiments in which participants experience and engage with actual natural and urban environments. As previously detailed, evidence from such studies comparing individual responses to natural and urban environments have offered evidence of benefits for affect and cognitive function, but less consistently through physiological measures. Indeed, the number of studies reporting physiological outcomes are relatively small and, not surprisingly given the greater logistical challenges, are often limited in terms of sample size, and the type and range of environments included (Bowler et al., 2010; Thompson Coon et al., 2011). As the equipment for field-based data collection has developed, so too has the potential for more ecologically valid experiments, including robust measures (e.g., HRV, cortisol). As such, the present research will add to the literature by investigating engagement with a natural environment on HRV and cortisol.

Social interaction within natural environments

There is substantial evidence from the social psychology literature that people have a fundamental need to belong, and forming social attachments is associated with increased well-being (Baumeister & Leary, 1995). Therefore, natural environments affording individuals greater opportunities for social interactions has been posited as a mediator of the natural environment-health relationship (Maas et al., 2009). This is based on the notion that opportunities for casual social contact are greater in neighbourhoods where residents spend more time in communal spaces, such as neighbourhood green spaces, in turn leading stronger social ties or social capital (Kuo, 2003; Kuo et al., 1998), with benefits for health (Gidlow et al., 2010; Poortinga et al., 2007).

Within this study, the nature and level of social contact within natural environment is of interest as a contributor to potential preventative and therapeutic effects. Few studies have explored how social interaction within natural environments influences individuals' affective, cognitive or physiological responses. Findings from an image-based study inferred that restorative benefits of walking might vary with social context (i.e., alone vs. in company) and the environment (forest vs. urban centre). Based on responses to questions relating to the attractiveness of walking in company or alone participants considered the forest walk better suited for restoration when alone (Staats & Hartig, 2004), with the presence of a friend associated with increased attractiveness of the urban walk. A later study used a similar, but field-based experiment, to explore differential affective and cognitive responses during 20-minute walks in urban parks and urban centres, with and without company (Johansson et al., 2011). Only revitalisation emerged as being moderated by environment and social context, increasing in park walks to a greater degree when alone, but increasing more during the walk along streets when with a friend. This is consistent with the nature of associations from the image-based study, but indicates an opportunity for further exploration.

Within this study, social interaction will be assessed during engagement with a natural environment to explore the role of and social interaction in individuals' responses.

Attitudes to the natural environment

In addition to the notions that restorative qualities of a natural environment can reduce stress (SRT) and promote restoration of depleted directed attention resources (ART), the degree to which an individual might benefit from natural environment exposure might depend on past experiences and personal connection to nature (Bratman et al., 2012). There is a suggestion that humans have an innate preference for natural, savannah-like environments (Falk & Balling, 2010; Kaplan & Kaplan, 1989), but that this is modified through experience and enculturation (Falk & Balling, 2010). Early childhood has been implicated as an important window during which experiences of environments can influence how we refer to them in later life (Adevi & Grahn, 2011). Furthermore, the frequency of visitation to certain types of environment has been linked to restorative potential, through individuals feeling 'at home' in more familiar surroundings (Adevi & Grahn, 2011). Such 'favourite' places may also have greater restorative potential (Korpela et al., 2010). Therefore, childhood experiences of natural environments are an important consideration.

Related to this (and perhaps as a result of early experiences) is how individuals vary in the extent to which they feel 'connected' or 'related' to nature, which may, in turn, moderate how they respond to different environments. In addition to making inferences based on environmental preferences, researchers have developed separate measures to quantify this human-nature connection. Scales that have been developed in the last decade include Connectedness to Nature Scale (Mayer & Frantz, 2004) and the Nature Relatedness Scale (Nisbet et al., 2009). There is some evidence of positive links with psychological and social well-being (Howell et al., 2011) and mixed results for emotional well-being (Cervinka et al., 2012; Howell et al., 2011; Mayer et al., 2008). There is also some indication that nature relatedness might moderate the positive affective responses to environments, with more 'nature related' people showing the greatest benefits from natural environment exposure, and greatest reductions in positive affect in alternative environments (Nisbet et al., 2009). However, this latter point is less studied and has been identified as an area for further work (Bratman et al., 2012), which will be explored in this study.

Environment Selection

There has been some criticism of the environmental selection in field-based studies. Not all have been designed to specifically test positive effects of natural environment exposure and, consequently, have not always included comparator sites to minimise the influence of potential confounders (Bowler et al., 2010; Bratman et al., 2012). For example, university campuses have been used as the natural environment, without sufficient description of the urban and natural qualities (Harte & Eifert, 1995). Others have included urban environments that are likely to have been considerably less 'pleasant' than the natural environments (Bodin & Hartig, 2003). Therefore, the possibility of effects arising from good versus bad environmental exposures, rather than natural versus urban, cannot be discounted in many cases. Several authors have called for investigations to consider more than one type of natural environment (Bowler et al., 2010; Bratman et al., 2012).

The aforementioned evidence that natural environments with water might be one of the most preferred, or offer greater benefits for affect (Barton & Pretty, 2010; Korpela et al., 2010; White et al., 2010), justifies comparisons between natural environments with/without the presence of water (e.g., lakes, rivers, streams) as a means of advancing this line of enquiry within this study, using a systematic process to select appropriate natural and comparator environments.

2. OBJECTIVES

- To explore therapeutic effects of natural environments.
- To evaluate the immediate and sustained changes in affective, cognitive and physiological responses indicative of well-being while engaged in a natural environment.
- To evaluate the longer-term changes in affective, cognitive and physiological responses indicative of well-being after leaving a natural environment.

3. METHODS

3.1. DESIGN

A randomised cross-over design will be used in which individuals (those with elevated stress) are exposed to different types of environments. The different types of environment will include green space (e.g., park like Collserola or Montjuïc), favourable blue space (e.g.,beach like Castelldefels), comparator urban environments (e.g., residential like Castelldefels town). The final exposure sites will be defined based on the results from an online survey. The online survey includs several images from different places and categories and asks to rate each image in how well it represents a certain type of environment.

Study participants (n=30) will be assigned to each environmental condition in a random order (to avoid ordering effects). Each volunteer will serve as its own control to exclude confounding by factors that are stable within an individual over time but vary between participants. To avoid a diurnal effect, all experiments and measurements will be scheduled equally during the day, always in weekdays. Exposure will involve transporting individuals to specific locations with given characteristics. Each volunteer is supposed to be exposed to all three environments. Exposure will be performed in groups of 6 people. The constituents of each group will be randomized for each exposure as much as possible. The experimental days of each participant will be at least 2 weeks apart but take part in the same season (between September and November 2013).

The exposure period will last 4,5 hours including lunch. However, taking into account exposure and pre and post measurement, participants will be involved in the study 8 hours each sampling day. During exposure, participants will be encouraged to do what they usually do in that environment, excluding vigorous physical activity. Eating and drinking will be controlled during the study period. Psychological and physiological responses will be measured using a standardised measures and protocols for consistency across partners.

Environmental exposures will be characterized using a standard approach developed by Phenotype group to maximise homogeneity of each environmental condition, but also to capture potentially important determinants. This will involve: audit to characterise environments prior to exposure and inform environment selection (audit tool will be identified/adapted); measurements during exposures (e.g., sunlight, noise, fine particulates, and NOx, social interaction). The latter, such as sunlight exposure, will be controlled during the exposures as much as possible.

Figure 1. Generic flow diagram of measurements and exposure

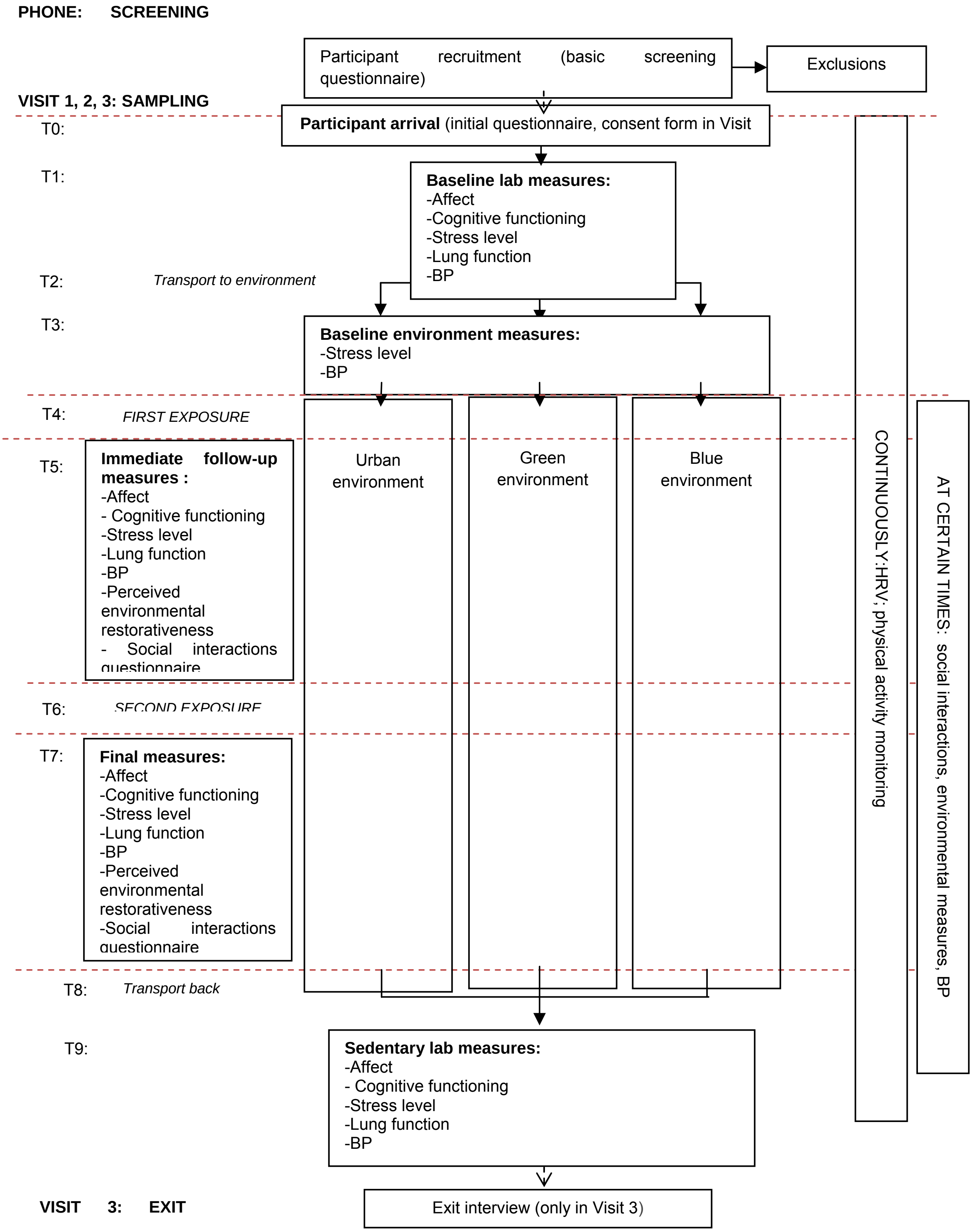

3.2. STUDY POPULATION

It is meant to include a representative/balanced group of volunteers in terms of age groups and sex, and natural environment exposure on daily life (distance to major natural areas (<300 m vs =>300 m)). The male to female ratio should be close to one.

Study participants (n=20-40) will be recruited from a study population of 1000 people living in Barcelona and recruited for WP2 of PHENOTYPE (age range 18-75). For WP2 of PHENOTYPE, all the 1000 people interviewed have been asked for their willingness to participate in other parts of PHENOTYPE project. From those willing to participate, the 10%ile with higher reported stress levels will be contacted randomly by phone and asked to participate in this study. After agreement, inclusion criteria will be checked. Recruitment process should start around one month before the sampling period.

Information about the project will be given to participants regarding the project (see Appendix 1). Before the enrolment in the study, all the participants will be asked to sign an informed consent (see Appendix 2).

<u>Inclusion criteria</u>

- From the highest 10%ile of our initial population based on reported stress levels.
- Between the ages of 18 – 75 years
- Non-smokers (at least one year prior to the study) (no smokers in the household if possible)
- Not pregnant determined by urine pregnancy test on all females
- Able to walk for 30 minutes at a self-directed pace
- Not chest or abdomen surgery during the last 3 months
- Not heart attack during the last 3 months
- Not hospitalized for heart problem during the last month
- Not retinal detachment or eyes surgery during the last month
- Pulse not higher than 120 bpm when in repose
- Not under tuberculosis treatment
- Not respiratory infection during the last three weeks
- Not asthmatic
- Without inhaler use during the last 24 hours.
- Not taking medications that could affect cortisol measures

3.3. DATA COLLECTION

At baseline participants will be required to complete a **<u>baseline profiling questionnaire</u>**. This questionnaire collects details relating to health and behaviour including (see Appendix 3): self-reported health, physical activity levels, life satisfaction, happiness with life, psychological well-being, attitudes towards natural environments (childhood exposure/enjoyment), information relating to the cognitive task.

HEALTH OUTCOMES

Cardiovascular monitoring

Heart Rate Variability (HRV): & Heart Rate (HR)

The monitoring of HRV and HR will start around one hour before exposure session (T1) and finish around one hour after (T9). HRV and HR will be monitored continuously using a Holter or eMotion unit. All subjects will be blinded to the results.

Blood Pressure:

Blood Pressure (BP) will be measured before exposure session (in the laboratory at T1 and in the environment at T3.), during the exposure (at some point during T6), at the end of the first exposure (immediate follow-up at T5), and at the end of the second exposure (final measures in the environment at T7 and sedentary lab measures at T9). BP will be measured using an automated blood pressure monitor. All subjects will be blinded to the results.

Physical activity level

The physical activity levels will start around one hour before exposure session (T1) and finish around one hour after (T9). Physical activity will be monitored continuously using a smartphone-based system called CalFit. The CalFit system runs on commercially available Android phones, allowing us to collect data on physical activity with accelerometry. Data from HR will also be used to assess physical activity.

Lung function

Lung function will be measured before exposure session (in the laboratory at T1), at the end of the first exposure (immediate follow-up at T5), and at the end of the second exposure (final measures in the environment at T7 and sedentary lab measures at T9). Lung function will be assessed with FEV1 (forced expiratory flow in 1 second) and FVC (forced vital capacity) measured using a portable computerized spirometer. The highest of three reading will be recorded at each time point.

Stress level

Stress level will be assessed before exposure session (in the laboratory at T1 and in the environment at T3.), at the end of the first exposure (immediate follow-up at T5), and at the end of the second exposure (final measures in the environment at T7 and sedentary lab measures at T9). Stress level will be assessed using salivary cortisol samples collected with Sarstedt salivettes (alternatively Salimetrics cortisol assay kit).

When analysing salivary cortisol it is important to consider the time-lag between external events and cortisol elevations (20-40 minutes) and also the possible factors that may influence cortisol levels. For that reason, it is important that baseline sample are taken in a consistent time after the arrival at PRBB (the initial idea is to take it just before leaving PRBB). Information on wake up time in the morning, oral contraceptive use and medication use will be obtained for female participants, and date of last period will be recorded (stage of the menstrual cycle can influence cortisol reactivity).

Affect

Affect will be assessed before exposure session (in the laboratory at T1), at the end of the first exposure (immediate follow-up at T5), and at the end of the second exposure (final measures in the environment at T7 and sedentary lab measures at T9). Affect will be assessed using the Brunel Mood Scale.

Cognitive functioning

Cognitive functioning will be assessed before exposure session (in the laboratory at T1), at the end of the first exposure (immediate follow-up at T5), and at the end of the second exposure (final measures

in the environment at T7 and sedentary lab measures at T9). Cognitive functioning will be assessed using the backwards digital span task (see Appendix 4).

Perceived Restoration

Perceived restoration will be assessed at the end of the first exposure (immediate follow-up at T5), and at the end of the second exposure (final measures in the environment at T7). Perceived restoration will be assessed using the Restoration Outcomes Scale (see Appendix 5).

EXPOSURE MONITORING

Environmental exposures in each area will be characterised using a standard approach. This will involve: audit to characterise environments prior to exposure and inform environment selection. It will also involve environmental measurements during exposures (e.g., temperature, humidity, sunlight, noise, fine particulates (DRX-CPC, and NOx).

Weather conditions

Temperature, air pressure, humidity and wind will be measured every 30 minutes during exposure.

Air pollution

Particulate matter

Ultrafine particles (UFP) will be assessed collecting particle number counts (PNC) with a real-time Condensation Particle Counter (Model 3007, TSI, MN, USA). The condensation particle counter uses 1-propanol as condensation liquid and measures particles larger than 10 nm (from 10 nm to 1000 nm in the concentration range of up to 105 Partikeln/cm3). Five second averages will be recorded.

Continuous particulate matter of the 2.5 fractions will be sampled using the real time monitor Dust Track (TSI) in a fixed point of each environment, each sampling day.

Black Carbon

The Black carbon (BC) and elemental carbon (EC) content in the air will be measured with a Micro-Aetholometer (McAgee) in a fixed point of each environment, each sampling day.

Carbon Oxides

Carbon Dioxide (CO2) and Carbon Monoxide (CO) will be measured using a Q-Track (TSI) in a fixed point of each environment, each sampling day.

Nitric Oxides

NOx air contents (NO and NO2 separately) will be monitored with a portable instrument (2B Technologies, model 401 and model 410) in a fixed point of each environment, each sampling day.

*N*oise

Noise levels (dba) will be measured via an sonometer (SC160- CESVA) located in a fixed point of each environment, each sampling day.

SOCIAL INTERACTIONS

At the end of the first exposure (immediate follow-up at T5), and at the end of the second exposure (final measures in the environment at T7) participants will be required to complete a short questionnaire about their social interactions during exposure (see Appendix 6).

Moreover, at certain times during all the sampling, research team will collect data about social interactions.

4. DATA MANAGEMENT

Data from many different data sources need to be combined. A separate data management protocol will be worked out. In general, questionnaires, biological samples (saliva) and measurements data will be safely stored in secure facilities, and names will be replaced by unique study numbers, and stored separately. Information will be accessed and handled by members of the research team only. Results will only be reported on a group level.

5. DATA ANALYSES

After data cleaning, descriptive analyses of the exposure (natural space characteristics), outcome (cardiovascular outcomes, lung function, stress level, affect/mood, cognitive functioning) and covariate data (e.g. gender, age, health status, physical activity, social interactions, air pollution, noise levels and temperature) will be performed.

Primary statistical analyses on the main natural space indexes (e.g. green/blue/urban environment, greenery, facilities) and outcomes will be performed with adjustment/stratification for relevant co-variates.

6. POTENTIAL RISKS

The participants will be exposed to a usual environment encountered during daily-life-activites, so no unusal risks are associated to this study. Selected places for the exposure will be places from Barcelona area usually visited for Barcelona citizens.

All measurements are non-invasive. All measurements will be conducted according to standard operating procedures. All staff working on the study will be properly trained.

Travelling to different environments is necessary as a quiet room and an exposure environment are needed to perform all the measurements each study day. First, volunteers will need to travel to the Parc de Recerca Biomèdica de Barcelona (PRBB) building at the beginning of the day, after it the six volunteers and three members from the research team will travel together to the exposure environment. After exposure, participants and research team members will travel back to PRBB. Travelling from PRBB to exposure environment and back will involve car rides.

6.1. INSURANCE

An insurance will be contracted. The insurance will cover potential costs of any consequence and claim derived from the study.

7. FINANCING

This project is financed by EU FP7-ENV-2008-1 Project number 227020.

8. PAYMENT OF PARTICIPANTS

The participants of the study will be paid 150 euros upon completion of the measurement campaign and checks for data quality for their participation.

9. REFERENCES

Adevi, A.A., & Grahn, P. (2011). Preferences for landscapes: a matter of cultural determinants or innate reflexes that point to our evolutionary background? Landscape research, 37(1), 27-49.

Appelhans, B. M., & Luecken, L. J. (2006). Heart rate variability as an index of regulated emotional responding. *Review of General Psychology*, *10*(3), 229–240. doi:10.1037/1089-2680.10.3.229

Barton, J., & Pretty, J. (2010). What is the best dose of nature and green exercise for improving mental health? A multi-study analysis. *Environmental science & technology*, *44*(10), 3947–55. doi:10.1021/es903183r

Bodin, M., & Hartig, T. (2003). Does the outdoor environment matter for psychological restoration gained through running? *Psychology of Sport and Exercise*, *4*(2), 141–153. doi:10.1016/S1469-0292(01)00038-3

Bornas, X., Llabrés, J., Noguera, M., López, A. M., Barceló, F., Tortella-Feliu, M., & Fullana, M. A. (2005). Looking at the heart of low and high heart rate variability fearful flyers: self-reported anxiety when confronting feared stimuli. *Biological psychology*, *70*(3), 182–7. doi:10.1016/j.biopsycho.2005.01.002

Bowler, D. E., Buyung-Ali, L. M., Knight, T. M., & Pullin, A. S. (2010). A systematic review of evidence for the added benefits to health of exposure to natural environments. *BMC public health*, *10*, 456. doi:10.1186/1471-2458-10-456

Bratman, G. N., Hamilton, J. P., & Daily, G. C. (2012). The impacts of nature experience on human cognitive function and mental health. *Annals of the New York Academy of Sciences*, *1249*, 118–36. doi:10.1111/j.1749-6632.2011.06400.x

Chang, C.-Y., Hammitt, W. E., Chen, P.-K., Machnik, L., & Su, W.-C. (2008). Psychophysiological responses and restorative values of natural environments in Taiwan. *Landscape and Urban Planning*, *85*(2), 79–84. doi:10.1016/j.landurbplan.2007.09.010

Gidlow, C., Cochrane, T., Davey, R. C., Smith, G., & Fairburn, J. (2010). Relative importance of physical and social aspects of perceived neighbourhood environment for self-reported health. *Preventive medicine*, *51*(2), 157–63. doi:10.1016/j.ypmed.2010.05.006

Gladwell, V. F., Brown, D. K., Barton, J. L., Tarvainen, M. P., Kuoppa, P., Pretty, J., Suddaby, J. M., et al. (2012). The effects of views of nature on autonomic control. *European journal of applied physiology*, *112*(9), 3379–86. doi:10.1007/s00421-012-2318-8

Hansen, A. L., Johnsen, B. H., & Thayer, J. F. (2003). Vagal influence on working memory and attention. *International Journal of Psychophysiology*, *48*(3), 263–274. doi:10.1016/S0167-8760(03)00073-4

Hartig, T., Evans, G. W., Jamner, L. D., Davis, D. S., & Gärling, T. (2003). Tracking restoration in natural and urban field settings. *Journal of Environmental Psychology*, *23*(2), 109–123. doi:10.1016/S0272-4944(02)00109-3

Hellhammer, D. H., Wüst, S., & Kudielka, B. M. (2009). Salivary cortisol as a biomarker in stress research. *Psychoneuroendocrinology*, *34*(2), 163–71. doi:10.1016/j.psyneuen.2008.10.026

Howell, A. J., Dopko, R. L., Passmore, H.-A., & Buro, K. (2011). Nature connectedness: Associations with well-being and mindfulness. *Personality and Individual Differences*, *51*(2), 166–171. doi:10.1016/j.paid.2011.03.037

Hug, S.-M., Hartig, T., Hansmann, R., Seeland, K., & Hornung, R. (2009). Restorative qualities of indoor and outdoor exercise settings as predictors of exercise frequency. *Health & place*, *15*(4), 971–80. doi:10.1016/j.healthplace.2009.03.002

Johansson, M., Hartig, T., & Staats, H. (2011). Psychological Benefits of Walking: Moderation by Company and Outdoor Environment. *Applied Psychology: Health and Well-Being*, *3*(3), 261–280. doi:10.1111/j.1758-0854.2011.01051.x

Kaplan, S. (1995). The restorative benefits of nature: toward an integrative framework. *Journal of Environ*, (15), 169–182.

Korpela, K. M., Ylén, M., Tyrväinen, L., & Silvennoinen, H. (2010). Favorite green, waterside and urban environments, restorative experiences and perceived health in Finland. *Health promotion international*, *25*(2), 200–9. doi:10.1093/heapro/daq007

Kuo, F. E., Sullivan, W. C., & Coley, R. L. (1998). Fertile Ground for Community□: Inner-City Neighborhood Common Spaces 1, *26*.

Laumann, K., Gärling, T., & Stormark, K. M. (2001). Rating Scale Measures of Restorative Components of Environments. *Journal of Environmental Psychology*, *21*(1), 31–44. doi:10.1006/jevp.2000.0179

Laumann, K., Gärling, T., & Stormark, K. M. (2003). Selective attention and heart rate responses to natural and urban environments. *Journal of Environmental Psychology*, *23*(2), 125–134. doi:10.1016/S0272-4944(02)00110-X

Maas, J., Dillen, S. Van, Verheij, R., & Groenewegen, P. (2009). Social contacts as a possible mechanism behind the relation between green space and health. *Health & place*, *15*, 586–595. doi:10.1016/j.healthplace.2008.09.006

Maas, J., Verheij, R. A., Groenewegen, P. P., Vries, S. De, & Spreeuwenberg, P. (2006). Green space, urbanity, and health: how strong is the relation? *J Epidemiol Community Health*, *60*, 587–592. doi:10.1136/jech.2005.043125

Mayer, F. S., & Frantz, C. M. (2004). The connectedness to nature scale: A measure of individuals' feeling in community with nature. *Journal of Environmental Psychology*, *24*(4), 503–515. doi:10.1016/j.jenvp.2004.10.001

Mitchell, R., & Popham, F. (2008). Effect of exposure to natural environment on health inequalities: an observational population study. *Lancet*, *372*(9650), 1655–60. doi:10.1016/S0140-6736(08)61689-X

Pretty, J., Peacock, J., Sellens, M., & Griffin, M. (2005). The mental and physical health outcomes of green exercise. *International journal of environmental health research*, *15*(5), 319–37. doi:10.1080/09603120500155963

Ryan, R. M., Weinstein, N., Bernstein, J., Brown, K. W., Mistretta, L., & Gagné, M. (2010). Vitalizing effects of being outdoors and in nature. *Journal of Environmental Psychology*, *30*(2), 159–168. doi:10.1016/j.jenvp.2009.10.009

Staats, H., & Hartig, T. (2004). Alone or with a friend: A social context for psychological restoration and environmental preferences. *Journal of Environmental Psychology*, *24*(2), 199–211. doi:10.1016/j.jenvp.2003.12.005

Staats, H., Kieviet, A., & Hartig, T. (2003). Where to recover from attentional fatigue: An expectancy-value analysis of environmental preference. *Journal of Environmental Psychology*, *23*(2), 147–157. doi:10.1016/S0272-4944(02)00112-3

Tennessen, C., & Cimprich, B. (1996). VIEWS TO NATURE□: EFFECTS ON ATTENTION. *Journal of Environmental Psychology*.

Thompson Coon, J., Boddy, K., Stein, K., Whear, R., Barton, J., & Depledge, M. H. (2011). Does participating in physical activity in outdoor natural environments have a greater effect on physical and mental wellbeing than physical activity indoors? A systematic review. *Environmental science & technology*, *45*(5), 1761–72. doi:10.1021/es102947t

Ulrich, R. S., Simons, R. F., Losito, B. D., Fiorito, E., Miles, M. a., & Zelson, M. (1991). Stress recovery during exposure to natural and urban environments. *Journal of Environmental Psychology*, *11*(3), 201–230. doi:10.1016/S0272-4944(05)80184-7

Van den Berg, A. E., Koole, S. L., & Van der Wulp, N. Y. (2003). Environmental preference and restoration: (How) are they related? *Journal of Environmental Psychology*, *23*(2), 135–146. doi:10.1016/S0272-4944(02)00111-1

White, M., Smith, A., Humphryes, K., Pahl, S., Snelling, D., & Depledge, M. (2010). Blue space: The importance of water for preference, affect, and restorativeness ratings of natural and built scenes. *Journal of Environmental Psychology*, *30*(4), 482–493. doi:10.1016/j.jenvp.2010.04.004

APPENDIX 1: INFORMATION FOR PARTICIPANTS

PHENOTYPE

Efectos positivos de la salud del medio ambiente natural en poblaciones típicas de diferentes lugares de Europa

¿Por qué el estudio PHENOTYPE?

La población que vive en ciudades está creciendo exponencialmente. Diferentes características de las ciudades pueden relacionarse con la salud de sus habitantes. Por ejemplo, la planificación de las ciudades, que determinará la movilidad de las personas y el nivel de acceso de estas a varios espacios, puede relacionarse con varios aspectos de la salud.

El proyecto PHENOTYPE se centra en las características, uso y acceso de la población a varios espacios de las ciudades.

¿Cómo funciona el estudio?

Algunas de las fases del proyecto PHENOTYPE son:

1) Obtención de datos de salud y de visita y uso de diversos espacios de las ciudades europeas a través de una encuesta a 1000 personas de cuatro ciudades europeas (incluyendo Barcelona) y de los datos de móviles y saliva de 100 personas de cuatro ciudades europeas (incluyendo Barcelona).

2) Medición de diferentes parámetros de salud de un grupo reducido de personas que visitaran determinados espacios bajo la supervisión del equipo investigador.

En este caso, le pedimos que participe en la segunda fase.

¿En qué consiste participar en la segunda fase?

Citaremos a los participantes un total de tres días para completar el estudio. Los citaremos de 9 de la mañana a 5 de la tarde, aproximadamente. Cada día de estudio habrá seis voluntarios acompañados por el equipo investigador.

Durante los días de estudio le pediremos que venga a nuestro centro por la mañana para realizarle una serie de pruebas y preguntas de salud. Una vez finalizadas estas pruebas iniciales le transportaremos, junto con otros cinco participantes, hasta un parque, una playa o una ciudad del área de Barcelona (el recorrido en coche será de unos 30 minutos). Después le pediremos que esté durante unas cuatro horas y media en este parque, playa o ciudad. Durante estas cuatro horas y media le realizaremos las mismas pruebas de salud que al inicio del día y le pediremos que vuelva a responder algunos cuestionarios. Después volveremos a transportarlo hasta nuestro centro para realizarle las mismas pruebas de salud y preguntas similares a las anteriores. Desde su llegado al centro hasta su partida al final del día, le pediremos que lleve un pequeño instrumento para medir su ritmo cardíaco y un móvil con una aplicación especial para registrar su actividad física.

Las pruebas de salud que le realizaremos incluirán: presión sanguínea, prueba de respiración en la que tendrá que respirar en un tubito y muestra de saliva. Las preguntas que le pediremos que responda incluirán preguntas sobre su salud, sus habilidades mentales, sus relaciones personales durante las horas del estudio y su percepción sobre determinados espacios.

En general, durante las cuatro horas y media en que esté en el parque, la playa o la ciudad podrá realizar las actividades que quiera. No obstante, le pediremos que no realice actividad física vigorosa y que no se bañe. También le daremos instrucciones sobre cuándo puede beber y comer durante los días de estudio. Los tres días de estudio le proporcionaremos el almuerzo.

¿Cuándo serán los días de estudio?

Los días de estudio serán durante los meses de setiembre, octubre y noviembre de 2013. Mayoritariamente serán los martes.

¿Qué beneficios obtendré si participo en la segunda fase del estudio PHENOTYPE?

Al final del estudio cada participante recibirá una remuneración de 150 euros por haber participado correctamente en los tres días de estudio. Así mismo, se le podrán enviar los resultados de las pruebas de salud realizadas si lo desea.

¿Qué sucede con la información recogida?

Las diferentes partes del estudio han estado aprobadas por el Comité Ético de Investigación Clínica del Parc de Salut Mar (CEIC-Parc de Salut Mar).

Toda la información recogida sobre usted durante el estudio será tratada por investigadores del CREAL de forma confidencial siguiendo lo que dicta la Ley Orgánica 15/99 de Protección de Datos de carácter personal. Así pues, toda su información estará identificada con un código para no poderse relacionar directamente con usted.

Todos los resultados del estudio se publicarán en revistas científicas y serán presentados en una base de datos del grupo del participante, no individualmente.

CENTRO DE INVESTIGACIÓN EN EPIDEMIOLOGIA AMBIENTAL (CREAL)
Parc de Recerca Biomèdica de Barcelona (PRBB)
C/Doctor Aiguader, 88, 08003, Barcelona
Tel: +34 214 73 00, e-mail: info@creal.cat

Personas de contacto:
Tània Martínez Íñiguez
Tel: 93 214 73 74, e-mail: tmartinez@creal.cat
Glòria Carrasco Turigas
Tel: 93 214 73 83, e-mail: gcarrasco@creal.cat
Margarita Triguero Mas
Tel: 93 214 73 79, e-mail: mtriguero@creal.cat

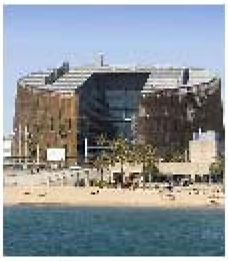

APPENDIX 2: CONSENT FORMS

centre de recerca en epidemiologia ambiental

CREAL

PHENOTYPE
Health from outside in

Estudio PHENOTYPE (WP4)
Hoja de consentimiento informado

Hoja de consentimiento para participantes mayores de edad en un proyecto de investigación biomédica

Efectos positivos en la salud del medio ambiente natural en poblaciones típicas de diferentes lugares de Europa.WP4: Efectos terapéuticos

Declaración del participante:

- Confirmo que he leído y entendido la información escrita y oral sobre el estudio que se me ha dado y que se me han aclarado satisfactoriamente todas las dudas posibles sobre el estudio. Declaro que conozco suficiente sobre los propósitos, métodos, ventajas e inconvenientes del estudio para decir que sí quiero participar.
- Entiendo y acepto que participo voluntariamente y que soy libre de abandonar el estudio en cualquier momento sin tener que dar una explicación, y sin que esto suponga que mi asistencia médica o mis derechos legales se vean afectados. Puedo solicitar que las muestras sean destruidas en cualquier momento, aunque los datos obtenidos hasta ese momento formarán parte del estudio.
- Se me ha informado del tratamiento confidencial de mis datos personales, según establece la Ley Orgánica 15/1999, de 13 de diciembre, de Protección de Datos de Carácter Personal.
- Doy mi consentimiento para participar en el proyecto de investigación PHENOTYPE y que para los datos recogidos sobre mi actividad física e información derivada de los cuestionarios esté guardados en una base de datos.
- Además, doy mi consentimiento para que mi material biológico (saliva) sea recogido y guardado en un banco de investigación médica para ser analizado en este estudio y en estudios futuros con objetivos similares de investigación. También, doy consentimiento para que se midan los parámetros del sistema cardiovascular (tensión, ritmo cardíaco) y parámetros pulmonares (capacidad pulmonar).
- Doy mi consentimiento para que todos los datos recogidos se usen para analizar los efectos positivos del medio ambiente natural en la salud de la población de Barcelona y, también, de Europa en general.
- Comprendo que investigadores del CREAL harán un uso estrictamente científico de todos los datos y muestras.
- Entiendo que para recibir la remuneración económica (150€) es necesario completar las tres fases del estudio correctamente.

En virtud de los artículos 4, 5 y 6 de la Ley Orgánica 15/1999 de 13 de diciembre, la *Fundació CREAL* pone en su conocimiento que dispone de un fichero con datos de carácter personal denominado *Investigación*.

La finalidad del fichero es desarrollar la investigación epidemiológica avanzada sobre factores ambientales que afectan a la salud humana, para facilitar la prevención y el control de sus efectos perjudiciales.

Los destinatarios de la información son el Grupo de Investigación encargado del estudio, así como los estamentos oficiales públicos o privados que, por obligación legal o necesidad material, deban acceder a los datos a efectos del correcto desarrollo del proyecto investigación, de acuerdo con las buenas prácticas científicas.

En cualquier caso, usted tiene derecho a ejercer los derechos de oposición, acceso, rectificación y cancelación en el ámbito reconocido por la Ley Orgánica 15/1999 de 13 de diciembre.

El responsable del fichero es la *Fundació CREAL*. Para ejercitar los derechos mencionados, y para cualquier aclaración, puede dirigirse por escrito mediante instancia dirigida a la Dirección de la Fundación en su domicilio sito en la calle Doctor Aiguader, 88, 08003 Barcelona.

Declaro que he recibido una copia de esta hoja de consentimiento y una copia de la información escrita sobre el proyecto.

Nombre del participante:………………………………………………………………………. ……………………………….

Fecha:…………………………………………….Firma: ……………………………………………………………………….

Desea que se le informe sobre los resultados de la presente investigación?

Sí |__| No|__|

Apreciamos sinceramente su cooperación en este proyecto de investigación.
Este protocolo ha sido revisado y aprobado por el Comité Ético de Investigación Clínica del Parc de Salut Mar (CEIC – PSMar).

APPENDIX 3: BASELINE PROFILING QUESTIONNAIRE

PERSONAL INFORMATION

Gender: Are you..... Male ☐ Female ☐

Date of Birth: ____________ (dd/mm/yyyy)

Including yourself, how many people live in your household?

Number of adults (16+ years): ____

Number of children (15 years or under): ____

Marital Status...Please tick the option that applies to you:

1. ...married / registered partnership	☐
2. living together	☐
3. with partner, but not cohabiting	☐
4. divorced / separated (but not divorced)	☐
5. unmarried / never married	☐
6. or widowed?	☐

Ethnicity...Please tick the option that best describes your ethnicity:

1. White	☐	
2. Mixed	☐	
3. Asian or Asian British	☐	
4. Black or Black British	☐	
5. Other	☐	Please specify:

Do you own a dog?
Yes ☐ No ☐

Have access to a car / van / motor bike for your personal use? Yes ☐ No ☐

Which of the following best describes your current work situation?

1. In Full-time work	☐
2. In Part-time work	☐
3. Casual	☐

4. Student/ in training	☐
4. Home duties	☐
5. Unemployed	☐
6. Retired	☐
7. Sickness leave	☐

What is your highest educational qualification?

1. Primary School	☐
2. Secondary School / Further Education (up to 16 years)	☐
3. Higher Education – University Degree or Higher (18+ years)	☐

Housing Tenure... Please tick the option that applies to you:

1. Own (outright)	☐
2. Own (with mortgage)	☐
3. Shard ownership (part own, part rent)	☐
4. Rent privately	☐
5. Rent social / local authority housing	☐
6. Rent free	☐

How long have you lived at your current address? _____ years _____ months

Are you a smoker?

1. Yes	☐
2. No, but I used to smoke	☐
3. No, I have never smoked	☐

Height ______ cm

Weight ______ kg

GENERAL HEALTH (SF12v2)

For each of the following questions, please select **one option** that best describes your answer.

1. In general, would you say your **health** is:

Excellent	Very good	Good	Fair	Poor
☐	☐	☐	☐	☐

2. To what degree does your health now **limit you in moderate activities,** such as moving a table, pushing a vacuum cleaner, bowling or playing golf?

Limited a lot	Limited a little	Not limited at all
☐	☐	☐

3. To what degree does your health now **limit you in climbing several flights of stairs**?

Limited a lot	Limited a little	Not limited at all
☐	☐	☐

4. During the past four weeks, how much of the time have you **accomplished less than you would like** in your work or other regular daily activities, as a result of your **physical health**?

All of the time	Most of the time	Some of the time	A little of the time	None of the time
☐	☐	☐	☐	☐

5. During the past four weeks, how much of the time have you been **limited** in the kind of work or other activities you do as a result of your **physical health**?

All of the time	Most of the time	Some of the time	A little of the time	None of the time
☐	☐	☐	☐	☐

6. During the past four weeks, how much of the time have you **accomplished less than you would like** in your work or other regular daily activities, as a result of **emotional problems** (such as feeling depressed or anxious)?

All of the time	Most of the time	Some of the time	A little of the time	None of the time
☐	☐	☐	☐	☐

7. During the past four weeks, how much of the time did you do work or other activities **less carefully than usual**, as a result of **emotional problems** (such as feeling depressed or anxious)?

All of the time	Most of the time	Some of the time	A little of the time	None of the time
☐	☐	☐	☐	☐

8. During the past four weeks, how much did **pain** interfere with your normal work, including both work outside the home and housework?

Not at all	Slightly	Moderately	Quite a bit	Extremely
☐	☐	☐	☐	☐

9. During the past 4 weeks, how much of the time have you felt **calm and peaceful**?

All of the time	Most of the time	Some of the time	A little of the time	None of the time
☐	☐	☐	☐	☐

10. During the past 4 weeks, how much of the time have you **had a lot of energy**?

All of the time	Most of the time	Some of the time	A little of the time	None of the time
☐	☐	☐	☐	☐

11. During the past 4 weeks, how much of the time have you felt **downhearted or depressed**?

All of the time	Most of the time	Some of the time	A little of the time	None of the time
☐	☐	☐	☐	☐

12. During the past 4 weeks, how much of the time has your **physical health or emotional problems interfered with your social activities** like visiting with friends, relatives etc?

All of the time	Most of the time	Some of the time	A little of the time	None of the time
☐	☐	☐	☐	☐

PHYSICAL ACTIVITY

Please take into your thought a **normal week** in the past months. Could you then indicate whether you are involved in the activities listed below and, if yes, how many days per week you were engaged in these activities, how much time you spent on it and with which average effort?

**Commuting from home to work and/or school**				
One answer per row; only for the respondents who work/go to school	No	Yes	Days per week	Time per day
a. Walking from/to work and/or school	☐	☐ →	___ days	___ hrs ___ min
b. Biking from/to work and/or school	☐	☐ →	___ days	___ hrs ___ min

**Physical activity at work and/or school**				
One answer per row	No	Yes	Days per week	Time per day
c. Light-moderate strenuous work (*sitting/standing with some walking, e.g. a desk job*)	☐	☐ →	___ days	___ hrs ___ min
d. Heavy strenuous work (*walking or lifting heavy stuff on a regular basis*)	☐	☐ →	___ days	___ hrs ___ min

**Household chores**				
One answer per row	No	Yes	Days per week	Time per day
e. Light to moderate strenuous work (*e.g. cooking, washing up, ironing*)	☐	☐ →	___ days	___ hrs ___ min
f. Heavy, strenuous work (*e.g. scrubbing floors, carry heavy groceries)*	☐	☐ →	___ days	___ hrs ___ min

**Leisure time**				
One answer per row	No	Yes	Days per week	Time per day
g. Walking	☐	☐ →	___ days	___ hrs ___ min
h. Bicycling	☐	☐ →	___ days	___ hrs ___ min
i. Gardening	☐	☐ →	___ days	___ hrs ___ min
j. Odd jobs	☐	☐ →	___ days	___ hrs ___ min
k. Sports	☐	☐ →	___ days	___ hrs ___ min
How important is it for you to do sports and/or to be physical active?	☐<br>☐<br>☐<br>☐<br>☐	Very important<br>Important<br>Somewhat important<br>Not important<br>Not important at all		

How much of your time being physically active is spent in a natural environment?	☐<br>☐<br>☐<br>☐<br>☐	0%<br>1-25%<br>26-50%<br>51-75%<br>75% or more

PERCEIVED STRESS

The questions in this scale ask you about your feelings and thoughts **during the last month**. In each case, you will be asked to indicate by circling *how often* you felt or thought a certain way.

**0**	**1**	**2**	**3**	**4**
Never	Almost Never	Sometimes	Fairly Often	Very Often

1. In the last month, how often have you been upset because of something that happened unexpectedly?	**0 1 2 3 4**
2. In the last month, how often have you felt that you were unable to control the important things in your life?	**0 1 2 3 4**
3. In the last month, how often have you felt nervous and “stressed”?	**0 1 2 3 4**
4. In the last month, how often have you felt confident about your ability to handle your personal problems?	**0 1 2 3 4**
5. In the last month, how often have you felt that things were going your way	**0 1 2 3 4**
6. In the last month, how often have you found that you could not cope with all the things that you had to do?	**0 1 2 3 4**
7. In the last month, how often have you been able to control irritations in your life?	**0 1 2 3 4**
8. In the last month, how often have you felt that you were on top of things?	**0 1 2 3 4**
9. In the last month, how often have you been angered because of things that were outside of your control?	**0 1 2 3 4**
10. In the last month, how often have you felt difficulties were piling up so high that you could not overcome them?	**0 1 2 3 4**

WELL-BEING: SATISFACTION WITH LIFE

Below are five statements that you may agree or disagree with. Using the 1 - 7 scale below, indicate your agreement with each item by placing the appropriate number on the line preceding that item. Please be open and honest in your responding.

**1**	**2**	**3**	**4**	**5**	**6**	**7**
Strongly disagree	Disagree	Slightly disagree	Neither agree or disagree	Slightly agree	Agree	Strongly agree

____ In most ways my life is close to my ideal.

____ The conditions of my life are excellent.

____ I am satisfied with my life.

____ So far I have gotten the important things I want in life.

____ If I could live my life over, I would change almost nothing.

SUBJECTIVE HAPPINESS

For each of the following statements and/or questions, please circle the point on the scale that you feel is most appropriate in describing you.

1. In general, I consider myself:

1	2	3	4	5	6	7
**not a very happy person**						**a very happy person**

2. Compared to most of my peers, I consider myself:

1	2	3	4	5	6	7
**less happy**						**more happy**

3. Some people are generally very happy. They enjoy life regardless of what is going on, getting the most out of everything. To what extent does this characterisation describe you?

1	2	3	4	5	6	7
**not at all**						**a great deal**

4. Some people are generally not very happy. Although they are not depressed, they never seem as happy as they might be. To what extent does this characterisation describe you?

1	2	3	4	5	6	7
**not at all**						**a great deal**

PSYCHOLOGICAL WELL-BEING

The following set of questions deals with how you feel about yourself and your life. Please indicate your degree of agreement on the scale below and please remember that there are no right or wrong answers.

1	2	3	4	5	6	7
Strongly agree	Somewhat agree	Agree a little	Neither agree or disagree	Disagree a little	Somewhat disagree	Strongly disagree

_____ I tend to be influenced by people with strong opinions.

_____ I have confidence in my own opinions, even if they are contrary to the general consensus.

_____ I judge myself by what I think is important, not by the values of what others think is Important.

_____ In general, I feel I am in charge of the situation in which I live.

_____ The demands of everyday life often get me down.

_____ I am quite good at managing the many responsibilities of my daily life.

_____ I think it is important to have new experiences that challenge how you think about yourself and the world.

_____ For me, life has been a continuous process of learning, changing, and growth.

_____ I gave up trying to make big improvements or changes in my life a long time ago.

_____ Maintaining close relationships has been difficult and frustrating for me.

_____ People would describe me as a giving person, willing to share my time with others.

_____ I have not experienced many warm and trusting relationships with others.

_____ I live life one day at a time and don't really think about the future.

_____ Some people wander aimlessly through life, but I am not one of them.

_____ I sometimes feel as if I've done all there is to do in life.

_____ When I look at the story of my life, I am pleased with how things have turned out.

_____ I like most parts of my personality.

_____ In many ways I feel disappointed about my achievements in life.

MOOD

Instructions: Below is a list of items that describe how people feel. There is no right or wrong answer so please answer as honestly as possible. For each item please tick the answer that best describes **how you feel right now**.

	Not at all	A little	Moderately	Quite a bit	Extremely
Panicky					
Lively					
Confused					
Worn-out					
Depressed					
Downhearted					
Annoyed					
Exhausted					
Mixed-up					
Sleepy					
Bitter					
Unhappy					
Anxious					
Worried					
Energetic					
Miserable					
Muddled					
Nervous					
Angry					
Active					
Tired					
Bad Tempered					
Alert					
Uncertain					

ATTITUDES TOWARDS NATURAL ENVIRONMENTS

Instructions: For each of the following, please rate the extent to which you agree with each statement, using the scale from 1 to 5 as shown below. Please respond as you really feel, rather than how you think "most people" feel.

**1**	**2**	**3**	**4**	**5**
Strongly disagree	Disagree a little	Neither agree or disagree	Agree a little	Strongly agree

____ 1. My ideal vacation spot would be a remote, wilderness area.

____ 2. I always think about how my actions affect the environment.

____ 3. My connection to nature and the environment is a part of my spirituality.

____ 4. I take notice of wildlife wherever I am.

____ 5. My relationship to nature is an important part of who I am.

____ 6. I feel very connected to all living things and the earth.

ENJOYMENT OF NATURE

Please rate below how much you would enjoy spending time visiting the following environments:

	Not<br>at all										Very Much
Green Space (e.g., Parks, Forests, Woodland, Countryside)	☐	☐	☐	☐	☐	☐	☐	☐	☐	☐	☐
Inland Water (e.g., Rivers, Lakes, Reservoirs, Canals)	☐	☐	☐	☐	☐	☐	☐	☐	☐	☐	☐
Coastal Areas (e.g., Oceans, Beaches)	☐	☐	☐	☐	☐	☐	☐	☐	☐	☐	☐
Mountains	☐	☐	☐	☐	☐	☐	☐	☐	☐	☐	☐
Other (please specify):	☐	☐	☐	☐	☐	☐	☐	☐	☐	☐	☐

CHILDHOOD EXPERIENCES OF NATURE

Please rate below how much time you spent visiting the following natural environments during your childhood:

	Not<br>at all										Very Much
Green Space (e.g., Parks, Forests, Woodland, Countryside)	☐	☐	☐	☐	☐	☐	☐	☐	☐	☐	☐
Inland Water (e.g., Rivers, Lakes, Reservoirs, Canals)	☐	☐	☐	☐	☐	☐	☐	☐	☐	☐	☐
Coastal Areas (e.g., Oceans, Beaches)	☐	☐	☐	☐	☐	☐	☐	☐	☐	☐	☐
Mountains	☐	☐	☐	☐	☐	☐	☐	☐	☐	☐	☐
Other (please specify):	☐	☐	☐	☐	☐	☐	☐	☐	☐	☐	☐

APPENDIX 4: COGNITIVE FUNCTIONING (BACKWARDS DIGIT SPAN TASK)

Practice Task

One point for each correct sequence. Stop after two consecutive errors.

No.	Read out:	Response	Correct?
1	9 2 6	6 2 9	
2	3 8 9	9 8 3	
3	5 1 9 3	3 9 1 5	
4	9 4 3 1	1 3 4 9	
5	1 8 6 3 4	4 3 6 8 1	

Cognitive **Task** **1**

One point for each correct sequence. Stop after two consecutive errors.

No.	Read out:	Response	Correct?
1	6 4 7	7 4 6	
2	7 9 6	6 9 7	
3	2 8 4 5	5 4 8 2	
4	8 1 3 7	7 3 1 8	
5	1 8 6 3 4	4 3 6 8 1	
6	7 2 9 8 4	4 8 9 2 7	
7	9 7 3 6 1 2	2 1 6 3 7 9	
8	6 2 8 3 1 7	7 1 3 8 2 6	
9	7 8 6 3 4 5 1	1 5 4 3 6 8 7	
10	3 5 1 8 4 2 7	7 2 4 8 1 5 3	
11	4 1 2 7 8 3 6 9	9 6 3 8 7 2 1 4	
12	1 8 5 4 7 9 6 2	2 6 9 7 4 5 8 1	
13	9 6 4 7 8 3 5 2 1	1 2 5 3 8 7 4 6 9	
14	5 6 7 2 1 9 3 4 8	8 4 3 9 1 2 7 6 5	
		**Total score**	

APPENDIX 5: RESTORATION OUTCOMES SCALE

Please read each statement carefully, then ask yourself, “How much does this statement apply to my experience here?”

To indicate your answer, circle one of the numbers on the scale.

	**Not at all**	**A little**	**Somewhat**	**Much**	**Very Much**
**1.** I feel calmer					
**2.** After visiting this place I feel restored and relaxed					
**3.** I have new enthusiasm and energy for my everyday routines from here					
**4.** My concentration and alertness clearly increased					
**5.** I forgot everyday worries here					
**6.** Visiting here cleared and clarified my thoughts					

APPENDIX 6: SOCIAL INTERACTIONS QUESTIONNAIRE

Since you arrived to this environment, how much time have you spent alone? ___h__min (approximately)

Since you arrived to this environment, how much time have you been with other people? (not including the research team)

___h__min (approximately)

Since you arrived to this environment, how much time have you been talking with other people? (not including the research team)

___h__min (approximately)

Since you arrived to this environment, how much time have you been enjoying talking with other people? (not including the research team)

___h__min (approximately)

Since you arrived to this environment, how much time have you been not feeling comfortable while talking with other people? (not including the research team)

___h__min (approximately)
